# Microbiota From Human Infants Consuming Secretors Mothers' Milk Affects Murine Intestinal Motility and Enteric Nervous System Development in a Sex‐Dependent Manner

**DOI:** 10.1111/nmo.70475

**Published:** 2026-11-04

**Authors:** Bharath K. Kumar, Valentina Caputi, Kirandeep K. Gill, Bindu Nanduri, Tanya Leroith, Renee Fox, Fernanda Rosa, Joshua M. Lyte, Laxmi Yeruva

**Affiliations:** ^1^ Microbiome and Metabolism Research Unit USDA‐ARS, Arkansas Children's Nutrition Center Little Rock Arkansas USA; ^2^ Department of Poultry Science Auburn University Auburn Alabama USA; ^3^ Poultry Production and Product Safety Research Unit Agricultural Research Service, USDA‐ARS Fayetteville Arkansas USA; ^4^ Department of Comparative Biomedical Sciences, College of Veterinary Medicine Mississippi State University Mississippi State Mississippi USA; ^5^ Department of Biomedical Sciences & Pathobiology Virginia Tech Blacksburg Virginia USA; ^6^ USDA‐ARS, Arkansas Children's Nutrition Center Little Rock Arkansas USA; ^7^ School of Veterinary Medicine Texas Tech University Amarillo Texas USA

## Abstract

**Background:**

Early life is a critical period in which the gut microbiome and the enteric nervous system (ENS) develop together, shaping long‐term gastrointestinal (GI) function. Human milk feeding and particularly maternal secretor phenotype strongly influence early microbiome composition. However, how human milk promoted microbiomes impact ENS development is still largely unknown.

**Methods:**

Male and female juvenile (three weeks old) C57BL/6 germ‐free mice were colonized with fecal microbiota from infants whose mothers were secretors (SMM, 2‐fucosyl lactose in milk) or non‐secretors (NSM) or from infants consuming cow's milk formula (MFM). Non‐transplanted male and female germ‐free mice served as controls. GI transit was assessed at post‐natal day 35‐37. ENS neuronal and glial organization was evaluated in full‐thickness colon preparations using immunohistochemistry and confocal microscopy.

**Key Results:**

Female germ‐free mice receiving SMM exhibited higher distal GI transit efficiency and a higher proportion of nitrergic neurons in the myenteric plexus compared to sex‐matched controls and to males. Choline acetyltransferase (ChAT; marker for excitatory cholinergic neurons) immunoreactivity was higher in the myenteric ganglia of germ‐free female compared to male mice. ChAT immunoreactivity increased in SMM and MFM males, while it was significantly reduced in NSM and MFM females compared to controls. Infant microbiomes did not affect myenteric glial cells, which were more abundant in males than females across all groups.

**Conclusions and Inferences:**

Our findings demonstrate that early‐life maternal secretor‐associated infant microbiomes exert sex‐specific effects on ENS neuronal phenotypes and GI motility, underlying a previously unrecognized link between infant‐derived microbiomes and ENS development that warrants investigation in larger cohorts.

## Key Points

- Infant microbiota from secretor mothers accelerate gastrointestinal transit efficiency in female juvenile mice.
- Microbiomes from infants fed by secretor mothers increase nitrergic neuronal populations within the myenteric plexus of female juvenile mice.
- Infant‐derived microbiomes modulate the myenteric cholinergic neuronal network in a sex‐dependent manner.
- Myenteric glial cells are more abundant in male than in female juvenile mice, and their number is not altered by infant microbiomes.

## Plain Language Summary

Our previous study shows that the type of milk infants receive—human milk from secretor [secretor gene influences human milk oligosaccharides composition by enabling production of 2′‐fucosyllactose (2′FL)] or non‐secretor mothers or cow's milk formula shapes their gut bacteria in ways that may influence gastrointestinal motility and the enteric nervous system. To test these effects, we transferred infant gut bacteria into young mice. While overall gut structure remained similar, the microbes affected males and females differently. In female mice, bacteria from infants fed by secretor mothers increased large‐intestinal motility and increased specific gut nerve cells (i.e., nitrergic neurons). These changes were not seen in males. Overall, our findings suggest that early feeding influences gut microbial communities in ways that can affect gut function later in life, with sex‐specific outcomes.

## Graphical abstract

Early feeding influences the gut microbiome, which programs gut neuronal function later in life. Transferring gut bacteria from breastfed and formula‐fed infants into germ‐free mice revealed sex‐specific effects. Microbiota from infants of secretor mothers increased transit efficiency and nitrergic neurons in the large intestine of female mice, while males showed no changes.

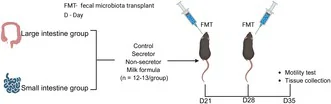

## 1 Introduction

The enteric nervous system (ENS) is a highly specialized neural network that regulates gastrointestinal (GI) motility, secretion, intestinal blood flow, and sensory transduction. Anatomically, the ENS encompasses 400 to 600 million neurons and glial cells that run along the entire length of the GI tract and are organized into two major ganglionated plexuses, one located within the muscularis externa (i.e., myenteric plexus), and the other one located within the submucosa (i.e., submucosal plexus) [1]. Although its foundational architecture is established during embryogenesis [2], ENS maturation extends through early postnatal life, a developmental window marked by robust neuroplasticity and heightened sensitivity to environmental cues, including the intestinal microbiome. Over the last decade, gut microbiota has emerged as a key regulator of ENS development. Microbial colonization begins during and immediately after birth, and these early colonizers provide metabolic, immune, and neuroactive signals that influence neuronal differentiation, glial maturation, synaptic organization, and the establishment of functional motility circuits [3].

Colonization patterns in early life are strongly influenced by feeding regimen (i.e., human milk or cow's milk formula). Human milk plays a central role in shaping the infant microbiome by providing a unique combination of beneficial bacteria and other nutritional factors, such as fatty acids and oligosaccharides that support healthy growth and neurodevelopment [4, 5]. In particular, human milk oligosaccharides promote the selective expansion of *Bifidobacterium* species, microbes that are typically found in lower abundance in infants fed with dairy formula, and higher in infants of secretor mothers [5]. Early‐life disruptions of microbiome‐host co‐developmental trajectories, arising from feeding patterns, maternal stress, antibiotics, or infections, can have long‐term consequences on GI function. Such perturbations are increasingly linked to the developmental origins of functional bowel disorders, characterized by intestinal dysmotility, visceral hypersensitivity, and altered gut–brain interactions [6]. Conditions including irritable bowel syndrome, functional dyspepsia, and infant colic have been associated with early alterations in the microbiota and impaired neuroimmune signaling [7], suggesting that disruptions in microbiome–ENS interactions during critical developmental windows may predispose individuals to persistent functional disorders.

Studies in germ‐free, gnotobiotic and antibiotic‐treated animal models demonstrate that disrupting early microbial exposure alters ENS architecture [8, 9, 10], impairs neuromuscular functions [11, 12], and causes lasting motility defects [13]. Despite this, direct evidence of ENS structural changes in human infants as a function of maternal secretor status, and how early‐life microbial environments shaped by maternal secretor phenotype might influence ENS maturation remains unexplored. Thus, addressing how early microbial environments shape ENS development is essential to developing early intervention strategies that support optimal enteric neuronal maturation and reduce susceptibility to early‐life induced GI dysfunction.

Maternal secretor status is determined by the expression of the fucosyltransferase 2 (FUT2) gene. FUT2 expression influences human milk composition, as secretor mothers produce high levels of 2′‐fucosyllactose (2′FL), a major fucosylated human milk oligosaccharide, whereas non‐secretor mothers produce little or no 2′FL. The impact of 2′FL‐containing milk is reflected in the developing infant gut microbiota. Specifically, Gurung et al. [4] demonstrated that infants fed by secretor mothers harbored an intestinal microbiota enriched in *Bacteroides*, *Parabacteroides*, and *Bifidobacterium* compared with infants fed by non‐secretor mothers [4].

The same study has shown that infant microbiomes shaped by maternal secretor status or formula feeding influence GI physiology and immune function when transplanted into juvenile germ‐free mice [4]. Since germ‐free mice display alterations in enteric neuronal density, glial structure, neurotransmission, and gut motility compared to conventionally raised animals, they serve as a powerful model for dissecting microbiota‐dependent mechanisms of ENS development. Building on these findings, we investigated how the infant microbiota programmed by maternal feeding status influences GI function and post‐natal ENS development, offering new insight into microbial drivers of early‐life enteric neuronal maturation.

## 2 Materials and Methods

### 2.1 Infant Fecal Samples Collection

The procedure of infant fecal sample collection was described in our previous publication [4]. Briefly, fecal samples were collected from 3–3.5‐month‐old human milk or dairy‐based formula fed infants that were enrolled in the Happy Tummies Study (clinical trial# NCT03493711) at the Arkansas Children's Nutrition Center. Parents collected fecal material at home from infants and stored at −20°C until transported to the research center where samples were stored at −80°C. Fecal samples were collected from healthy infants who had not received antibiotics for at least two weeks. Maternal milk samples were used to determine secretor status in Dr. Lars Bode's laboratory at the University of California‐San Diego (UCSD) based on 2′‐fucosyllactose (2′‐FL) concentration, as published previously [4]. Fecal material from infants fed with secretor (*n* = 5), non‐secretor (*n* = 5) human milk, or formula (*n* = 5) was pooled by group. Dairy‐based formula‐fed infants received Similac Advance with prebiotics galacto‐oligosaccharides (GOS) (Abbott Nutrition). For each pooled sample, 100 mg of fecal material was mixed with 1 mL reduced 1X Phospate Buffered Saline (PBS) containing 0.10% resazurin and 0.05% L‐cysteine. Preparations were vortexed and settled for 5 min before collecting the supernatant, which served as the microbiota inoculum for germ‐free mouse transplantation.

### 2.2 Fecal Transplantation of Human Feces to Germ‐Free Mice

The Institutional Animal Care and Use Committee at the University of Arkansas for Medical Sciences approved the experimental procedures used in this study. Female and male C57BL/6 germ‐free mice at 21–23 days of age were randomized into two experimental groups, based on body weight. Within each experimental group, mice were further randomly distributed across four treatment groups (*N* = 10–13 per group) based on the bodyweight: (1) fecal microbiota transplants from infants of secretor mothers (SMM), (2) fecal microbiota transplants from infants of non‐secretor mothers (NSM), (3) fecal microbiota transplants from infants fed milk formula (MFM), and (4) vehicle gavage without fecal transplantation (Control). The experimental period lasted for 14 consecutive days. During the experimental period, on Days 1 and 7, each mouse within the treatment groups received 100 μL of fecal inoculum of respective transplants (total amount 10 mg feces/mouse). The control group received 100 μL of sterile PBS. At post‐natal day (PND) 35–37 (end of the experiment), fresh fecal samples were collected for 16S rRNA sequencing and microbiome analysis. Gastrointestinal motility assessments were performed in two separate experiments, as described below, and after that, mice were euthanized by cervical dislocation.

### 2.3 16S rRNA Gene Sequencing and Bioinformatics

DNA was extracted from the fecal samples collected on PND 35–37 as described previously [4]. The target region amplified for 16S was V3‐V4, using primers 341F (CCTAYGGGRBGCASCAG) and 806R (GGACTACNNGGGTATCTAAT). The amplicon sequencing reads for 16S rRNA were obtained from Novogene as de‐multiplexed reads with primers trimmed. Paired end joining of the forward and reverse reads was done in QIIME2 version 2024.5.1 using the default parameters. The sequencing reads were then classified to phylogenetic levels using SILVA bacterial reference database version 138.2 at a similarity threshold of 99%. The taxonomy file and the feature table were then converted to a phyloseq object in R (version 4.4.1). We used MicrobiomeAnalyst, a web‐based platform, to determine α‐ and β‐diversity (Bray–Curtis dissimilarity) and taxon abundances from the OTU tables (parameters: minimum count of 5, rarefied data; prevalence in the sample of 20%, percentage to remove of 10%, and data scaling set to total sum scaling).

### 2.4 Gastrointestinal Transit

GI motility was assessed by using a non‐absorbable fluorescent tracer, fluorescein isothiocyanate–dextran (FITC)‐labeled dextran (250 kDa; Sigma‐Aldrich FD250S) [13]. Female and male mice were orally gavaged with FITC‐dextran (6.25 mg/mL dissolved in 0.9% saline; 100 μL/mouse). Motility was assessed in two separate experiments, as described previously [14]. In *Experiment 1*, mice were euthanized 40 min post‐gavage, and in *Experiment 2*, mice were euthanized 180 min (3 h) post‐gavage. Each experiment was conducted over two days within the same circadian window, with mice divided into two cohorts (*Experiment 1*: *N* = 3/group/day/cohort; *Experiment 2*: *N* = 3–4/group/day/cohort). At the end of the 40 min (*Experiment 1*) and 180 min (*Experiment 2*), mice were euthanized (anesthesia by isoflurane followed by cervical dislocation), and the GI tract was carefully removed. After recording the length of the small and large intestine, the GI tract was divided into 9 segments in *Experiment 1* (one stomach segment [Sto], 6 equal‐length segments of small intestine [SI1‐SI6], one caecum segment [Cecum], and one colon segment) and 11 segments in *Experiment 2* (one stomach segment [Sto], 6 equal‐length segments of small intestine [SI1‐SI6], one caecum segment [Cecum], and 3 equal‐length colon segments [Col1‐Col3]). Luminal contents from each segment were collected and centrifuged at 10,000 × g for 10 min at 4°C. The fluorescence intensity of FITC dextran was measured in the supernatants at 492/521 nm using a microplate reader (BMG Labtech, Cary, NC). The transit of FITC dextran along the GI tract was determined by calculating the percentage fluorescence for each segment and expressed as geometric center (GC) using the following equation: GC = Σ (% of total fluorescence signal × segment * segment number)/100 [11]. Tissue segments of small intestine (SI1 = jejunum and SI5 = ileum), cecum, and full‐length colon were collected from mice during *Experiment 1*. SI1, SI5, and cecum were fixed in 10% formalin for histomorphometric analysis, while the colon was used for immunostaining. Colon segments for histomorphometric analysis were collected during *Experiment 2* and fixed in 10% formalin.

### 2.5 Histomorphometric Analysis

Briefly, formalin‐fixed tissues (SI1, SI5, cecum and colon) were paraffin‐embedded, cut to 5‐μm thickness, and stained with hematoxylin and eosin. Crypt depth and villus height were measured using the pen tool in Aperio ImageScope software, capturing 10 representative images per mouse with selected crypts adjacent to the measured villi. A board‐certified pathologist, Dr. Tanya LeRoith, performed the morphological analysis in a blinded manner as previously described [4].

### 2.6 Immunohistochemistry and Tissue Clearing

Colon tissues were filled with fixative solution 4% paraformaldehyde in 10 mM PBS for 3 h at 4°C in a 50 mL conical tube [15]. Colon segments were then cut into 1 cm pieces and opened along the mesenteric border. Flat colon tissues were rinsed in 10 mM PBS and incubated in blocking solution containing 5% Normal Horse Serum, 5% Bovine Serum Albumin, 0.75% Triton X‐100 in 10 mM PBS for 20 h at room temperature (RT). Colon tissues were incubated with the primary antibodies (Table S1) diluted in the blocking solution for 24 h (overnight) at 4°C. Tissues were then washed with washing buffer (10 mM PBS containing 0.05% Triton X‐100 and 0.5% 1‐thioglycerol) and incubated with the secondary antibodies (Table S1) diluted in 10 mM PBS + 0.5% Triton X‐100. Double and triple labelling were performed during consecutive incubation times, as described in the Supporting Information. Colon tissues were washed 5 × 30 min at RT with washing buffer and subjected to aqueous‐based clearing procedure, as previously described [16].

### 2.7 Confocal Imaging and Image Analysis

Colon tissues were imaged with a Leica Stellaris 5 laser scanning confocal microscope (Leica, Houston, TX, US), equipped with a HC PL APO CS2 10X/0.40 dry objective. Multi‐tiles Z‐stack images (20–40 tiles, 20–25 z‐steps of 2.4 μm each) of 512 × 512 pixels were captured and processed as maximum intensity projections. All microscope settings were set to collect images below saturation and were kept consistent for all images (see Supporting Information).

The number of HuC/D^+^ or HuC/D^+^nNOS^+^ neurons was computed and normalized to the myenteric ganglion area (μm^2^; 15–20 ganglia/image/mouse), as previously described [17]. Boundaries of myenteric ganglia were determined as locations where the distance of two agglomerates of cells was less than the average Feret diameter of one neuronal cell [18]. Changes in choline acetyltransferase (ChAT) and substance *P* immunoreactivities were assessed by analyzing the density index of ChAT^+^ or substance *P* ^+^ labeling for each myenteric ganglion area (μm^2^; 8–10 ganglia/image/mouse; Supporting Information). Changes in enteric glia populations and glia‐to‐neuron ratio were assessed by counting GFAP^+^‐S100β^+^ cell bodies (glia) and HuC/D^+^ cell bodies (neurons) in each myenteric ganglia area (μm^2^; 8–10 ganglia/image/mouse), as previously described [19]. Image analyses were performed in a blinded manner using FIJI software (Fiji is just Image J, version 1.54p).

### 2.8 Statistical Analysis

Data were expressed as mean ± standard error of the mean (SEM). Normality was assessed with Shapiro–Wilk and Kolmogorov–Smirnov tests. Homogeneity of variance was evaluated with the Bartlett and Brown‐Forsythe tests. Data were analyzed by two‐way ANOVA followed by Tukey's multiple comparison tests in GraphPad Prism (version 11.0). Infant feeding status (e.g., microbiota) and sex were included as the main effects. Differences were considered statistically significant at *p* < 0.05 for both main effects and their interaction.

## 3 Results

### 3.1 Infant Feeding Status Influences the Fecal Microbiome Composition Without Affecting the Structural Morphology of the Gut Mucosa

In agreement with the previous publication by Gurung et al. [4], Bray–Curtis dissimilarity method revealed that infant feeding status results in distinct clustering in fecal microbiome composition, with PCoA 1 and PCoA 2 axes explaining 34% and 42% of the total variance, respectively (PERMANOVA R2 = 0.93, *p* = 0.001; panel (I), Figure 1A). Additionally, we performed PERMDISP analysis to determine whether these differences were due to within‐group variability. The results revealed that PERMDISP was significant (*p* = 0.001; panel (II), Figure 1A), indicating that dispersion differed among groups. Therefore, PERMANOVA significance may reflect both community differences and within‐group variability. However, these differences are expected in fecal microbiome transplant studies because individual mice may differ in their microbiome composition due to differences in the engraftment of the transferred microbiota.

**FIGURE 1:**
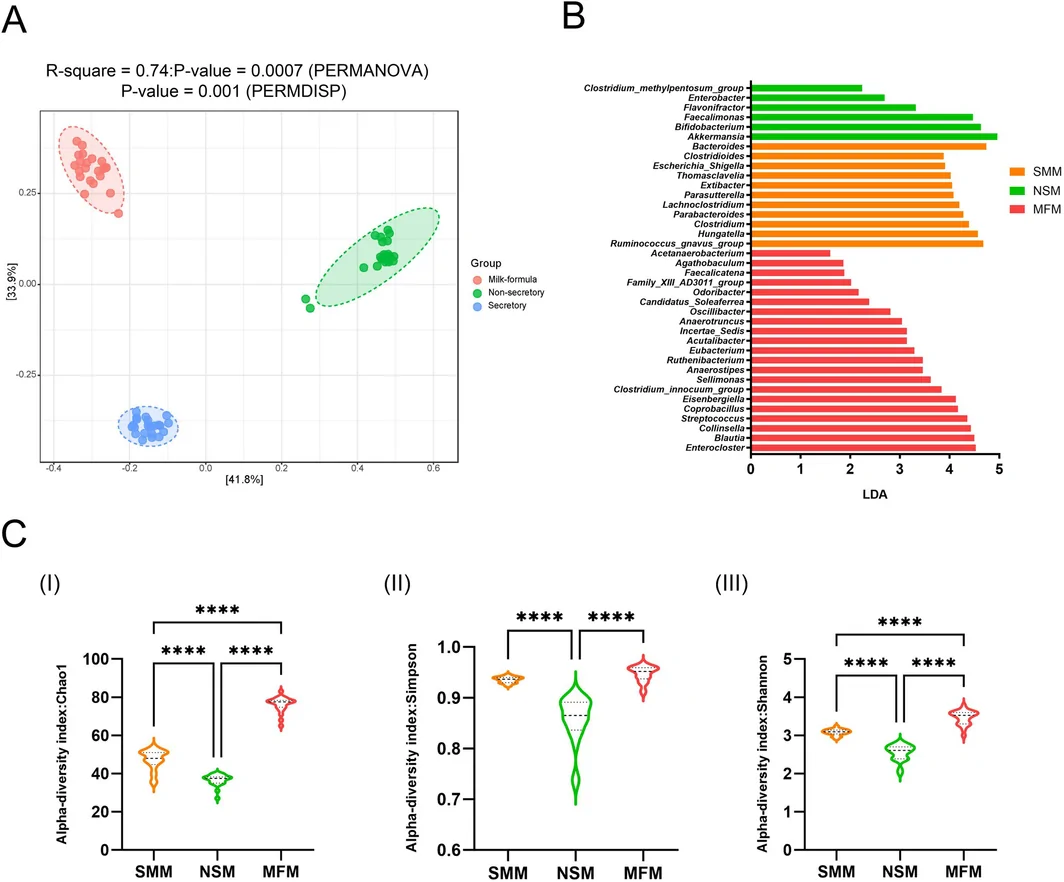
Infant feeding status influences the fecal microbiome composition. (A) Dissimilarity in gut microbiome composition among infant feeding groups as shown by Bray–Curtis β‐diversity. Group differences in β‐diversity were assessed using permutational multivariate analysis of variance (PERMANOVA) and within‐group variation was evaluated using PERMDISP. (B) Differentially abundant bacterial genera among mice gavaged with SMM, NSM, and MFM were identified using linear discriminant analysis effect size (LEfSe; FDR5 < 0.05). (C) α‐diversity as shown by Chao1 (I), Simpson (II), and Shannon (III) indices. β‐diversity, α‐diversity, and LEfSe analysis were performed in MicrobiomeAnalyst (2.0). SMM = mice transplanted with fecal material from infants consuming milk from secretor mothers, NSM = mice transplanted with fecal material from infants consuming milk from non‐secretor mothers, MFM = mice transplanted with fecal material from infants consuming dairy‐based formula (*N* = 20–23 mice/group). *p ≤ 0.05, **p ≤ 0.01, ***p ≤ 0.001, ****p ≤ 0.0001.

Further, Linear Discriminant Analysis Effect Size (LEfSe) analysis identified the microbial taxa that discriminated among feeding groups (Figure 1B). According to the LEfSe analysis, the SMM group was enriched for genera such as *Bacteroides, Hungatella*, and *Ruminococcus*. In contrast, the NSM group was enriched for genera such as *Bifidobacterium* and *Akkermansia*, and the MFM group was enriched for genera such as *Blautia*, *Enterocloster*, and *Collinsella* (Figure 1B). Further, MFM had significantly higher alpha‐diversity than the SMM or NSM groups (Figure 1C). Notably, the effects of infant feeding status on α‐diversity, β‐diversity, and microbial taxa identified by LEfSe were consistent between male (Figure S1) and female mice (Figure S2).

As shown in Figure S3, villus height and crypt depth in the upper (SI1) and lower (SI5) small intestine, as well as gland depth in the cecum and colon, did not differ among groups.

### 3.2 The Microbiota of Maternal Milk or Dairy Formula Fed Infants Does Not Influence Gastrointestinal Progression of Luminal Content in the Small Intestine

To assess whether SMM, NSM, or MFM influence intestinal motility, we evaluated the distribution of a non‐absorbable FITC–dextran fluorescent marker throughout the GI tract of juvenile mice at both an early (40 min) and a later (180 min) time point following oral administration. A two‐way ANOVA revealed a significant difference in the distribution of FITC dextran fluorescence across the different segments of the GI tract (*P*_Intestinal segments_ < 0.0001) in both male (Figure 2A) and female (Figure 2B) mice. Tukey's multiple comparisons between intestinal segments (within each treatment group) revealed a significant increase of FITC–dextran fluorescence in SI4 compared to the other segments (Sto, SI1,2,3,5, 6, Cecum and colon) in both male (Table S2) and female (Table S3) mice. In male mice, a significant increase in FITC dextran fluorescence (*p* < 0.05) was also observed in SI3 compared to SI1, 2, 6, Sto, cecum and colon in the NSM group (Table S2), suggesting a slower motility in the NSM group compared to the others. Tukey's multiple comparisons between treatment groups showed a significant decrease in FITC dextran fluorescence in SI5 (ileum) of female SMM compared to control mice (*p* < 0.05; Figure 2B), suggesting a slower progression of the luminal content in the SMM group in the distal end of the small intestine.

**FIGURE 2:**
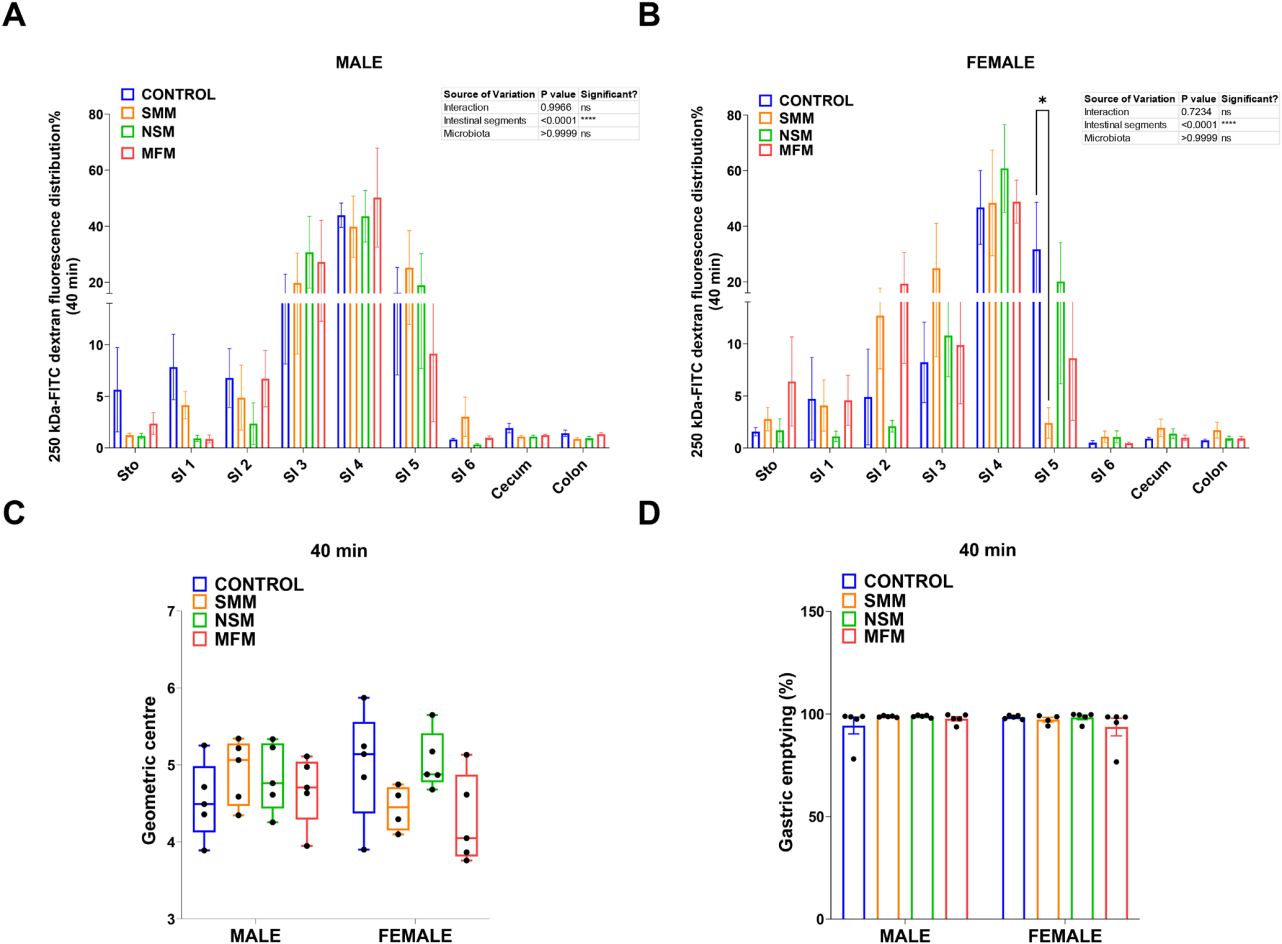
Microbiota of infants consuming milk from secretor or non‐secretor mothers or infant formula does not affect GI transit in the small intestine of juvenile mice. Percentage of non‐absorbable 250‐kDa FITC dextran distribution along the GI tract consisting of stomach (Sto), small intestine (SI1–S6), cecum and colon in male (A) and female (B) mice, measured 40 min after fluorescent marker gavage. (C) Geometric center of non‐absorbable 250‐kDa FITC dextran distribution and (D) percentage of gastric emptying in the different experimental groups. Data are reported as mean ± SEM for panels (A), (B) and (D), and as median, minimum, maximum, upper and lower quartiles for panel (C). The effect of microbiota, intestinal segments and their interactions were determined using a two‐way ANOVA with Tukey's multiple comparison test in GraphPad Prism Version 11 (www.graphpad.com). (A) and (B) *P*_intestinal segments_ < 0.0001. (B) *p < 0.05 SMM vs. control. SMM = mice transplanted with fecal material from infants consuming milk from secretor mothers, NSM = mice transplanted with fecal material from infants consuming milk from non‐secretor mothers, MFM = mice transplanted with fecal material from infants consuming dairy‐based formula, CONTROL = germ‐free mice. (*N* = 4–5 mice/sex/group).

The geometric center (GC) is a quantitative measure that helps determine how far the contents have moved from the stomach into the small intestine. A two‐way ANOVA analysis revealed no effect of maternal secretor status or dairy‐based formula consumption on the GC of male or female germ‐free mice (Figure 2C). Male and female mice of all treatment groups exhibited almost complete gastric emptying after 40 min of marker gavage (98%–99%), with no differences between groups (Figure 2D).

### 3.3 The Microbiota From Infants Fed by Secretor Mothers Increases the Gastrointestinal Progression of Luminal Contents in the Large Intestine of Female Mice

The distributions of FITC dextran fluorescence throughout the GI tract after 180 min of oral gavage are shown in Figure 3. In both sexes, the peaks of the marker fluorescence were observed mainly in the cecum. In male mice, there were no differences in the distribution of FITC dextran fluorescence between the 3 treatments and the control group (Figure 3A and Table S4). Conversely, an increased progression of the luminal contents in the 3 treatment groups (SMM, NSM, MFM) compared to the controls was observed in female mice, as shown in Figure 3B (two‐way ANOVA *P*_interaction_ < 0.05; *P*_intestinal segments_ < 0.0001). In particular, FITC dextran content was significantly higher in SI5 and SI6 in control mice compared to SMM (SI5 = *p* < 0.05; SI6 = *p* < 0.01) and NSM (SI6 = *p* < 0.01) mice (Figure 3B); however, in the cecum, FITC dextran fluorescence was lower in the control mice compared to the SMM mice (*p* < 0.01; Figure 3B). Tukey's ‘multiple comparisons between intestinal segments (within each treatments group) in female mice revealed a significant increase of FITC dextran fluorescence in SI6 and cecum compared to the other segments within the control group (Table S5); however, in the SMM group, the fluorescence in the luminal content of cecum and colon 1 was significantly higher compared to the other segments within the SMM group (Table S5). In agreement with these findings, the GC of SMM female mice (GC_SMM‐F_ = 8.389) was higher than the control mice (GC_CONTROL‐F_ = 6.723), although not statistically significant (*p* = 0.063) (Figure 3C). No differences in gastric emptying were observed between groups (Figure 3D).

**FIGURE 3:**
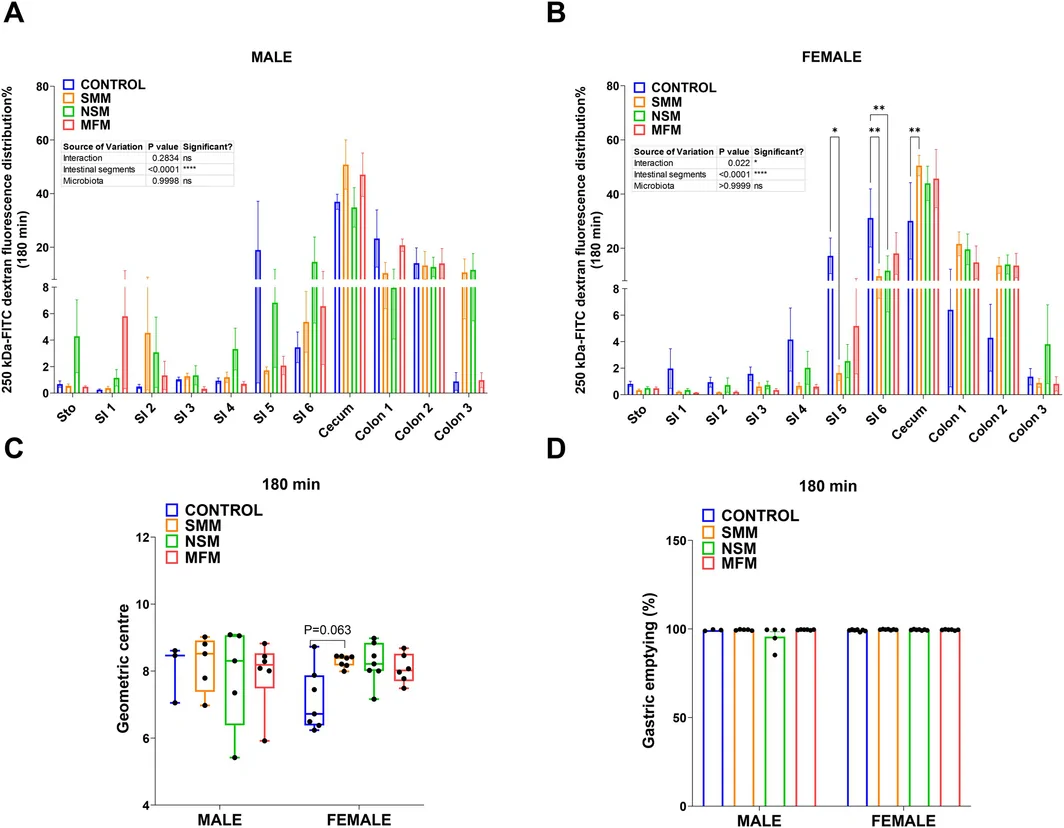
Microbiota from infants fed by secretor mothers accelerates intestinal motility in the large intestine of female mice. Percentage of non‐absorbable 250‐kDa FITC dextran distribution along the GI tract of male (A) and female (B) mice, measured 180 min after fluorescent marker gavage. In these experiments, the GI tract consisted of stomach (Sto), small intestine (SI1–S6), cecum and Colon 1–3. (C) Geometric center of non‐absorbable 250‐kDa FITC dextran distribution and (D) percentage of gastric emptying in the different experimental groups. Data are reported as mean ± SEM for panels (A), (B) and (D) and as median, minimum, maximum, upper and lower quartiles for panel. The effect of microbiota, intestinal regions and their interactions were determined using a two‐way ANOVA with Tukey's multiple comparison test in GraphPad Prism Version 11 (www.graphpad.com). (A) *P*_Intestinal segments_ < 0.0001. (B) *P*_Intestinal segments_ < 0.0001; *P*_Interaction_ < 0.05; *p < 0.05 and **p < 0.01 SMM vs. control; **p < 0.01 NSM vs. control. SMM = mice transplanted with fecal material from infants consuming milk from secretor mothers, NSM = mice transplanted with fecal material from infants consuming milk from non‐secretor mothers, MFM = mice transplanted with fecal material from infants consuming dairy‐based formula. CONTROL = germ‐free mice. (*N* = 3–7 mice/sex/group).

### 3.4 The Microbiota of Infant Fed by Secretor Mothers Increases the Nitrergic Neuronal Population in the ENS of Female Mice

We assessed how infant microbiota shaped by maternal secretor status or formula feeding affects ENS structure in the colon using immunofluorescence. HuC/D served as a pan‐neuronal marker for the cell bodies of all enteric neurons [20, 21], while nNOS together with HuC/D (nNOS^+^HuC/D^+^) was used to identify the subpopulation of enteric nitrergic neurons (i.e., inhibitory motor neurons and descending interneurons [22]). A two‐way ANOVA revealed significant interaction effects on the density of HuC/D^+^ (*P*_interaction_ = 0.0098), and nNOS^+^HuC/D^+^ neurons (*P*_interaction_ = 0.0243) per ganglion area in the colonic myenteric plexus (Figure 4A–D).

**FIGURE 4:**
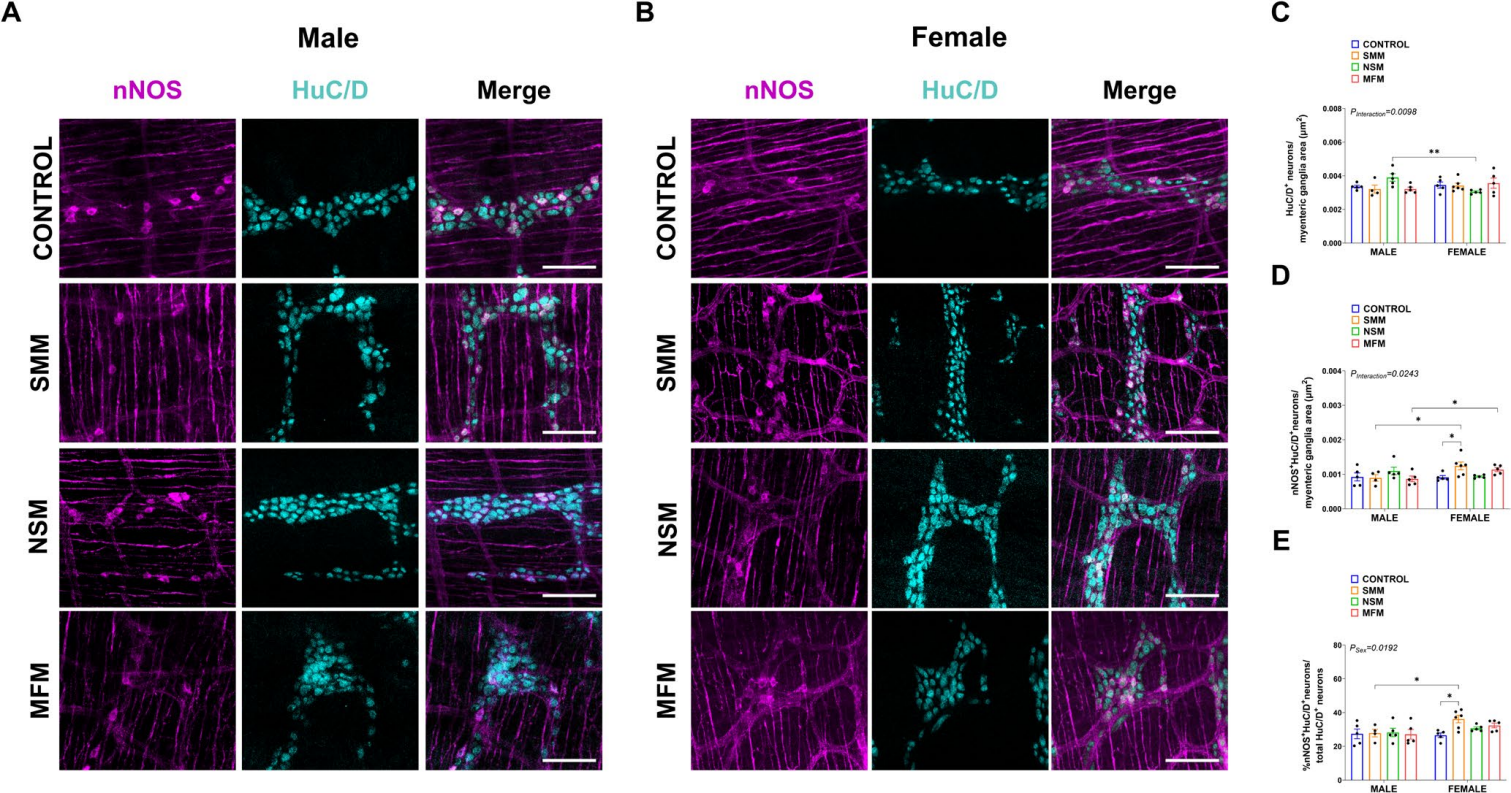
Effect of microbiota from infants fed by secretor mothers on the nitrergic neuronal population in the myenteric plexus of juvenile mice. Representative confocal photomicrographs showing the distribution of nNOS (magenta, marker for nitrergic neurons) and HuC/D (cyan, pan‐neuronal marker marker) in the myenteric plexus of male (A) and female (B) mouse colon. Scale bars = 100 μm. Quantification of the number of HuC/D^+^ neurons (C), nNOS^+^‐HuC/D^+^ neurons (D), and percentage of nNOS^+^‐HuC/D^+^ neurons in the myenteric ganglia of male and female mice. Data are reported as mean ± SEM for panels (C), (D), and (E). The effect of microbiota, sex and their interactions were determined using a two‐way ANOVA with Tukey's multiple comparison test in GraphPad Prism Version 11 (www.graphpad.com). (C) **p < 0.01 male NSM vs. female NSM. (D) *p < 0.05 female SMM vs. female control and vs. male SMM; *p < 0.05 female MFM vs. male MFM. (E) *p < 0.05 female SMM vs. female control and vs. male SMM. SMM = mice transplanted with fecal material from infants consuming milk from secretor mothers, NSM = mice transplanted with fecal material from infants consuming milk from non‐secretor mothers, MFM = mice transplanted with fecal material from infants consuming dairy‐based formula, CONTROL = germ‐free mice. (*N* = 4–6 mice/sex/group).

Tukey's multiple comparisons test showed that male NSM mice had significantly higher density of HuC/D^+^ neurons than group‐matched females (*p* < 0.01; Figure 4A–C). The density of nNOS^+^HuC/D^+^ neurons was significantly higher in the female SMM (*p* < 0.05) and MFM (*p* < 0.05) mice compared to group‐matched male mice (Figure 4A,B,D). Moreover, the density of nNOS^+^HuC/D^+^ neurons was significantly increased in the female SMM mice compared to sex‐matched controls (*p* < 0.05; Figure 4B,D). We also calculated the percentage of nNOS^+^HuC/D^+^ neurons on the total of HuC/D^+^ neurons, which was significantly higher in female SMM compared to sex‐matched controls (*p* < 0.05) and to group‐matched male mice (*p* < 0.05), as shown in Figure 4E.

### 3.5 Microbiota of Infants Fed With Maternal Milk or Dairy Formula Affects the Excitatory Neuronal Components of the ENS in a Sex‐Dependent Manner

The myenteric plexus contains two major excitatory neuronal networks that drive smooth muscle contraction during peristalsis. Cholinergic neurons release acetylcholine, acting on muscarinic receptors to stimulate contraction. Tachykinergic neurons release substance P, which enhances smooth muscle contraction and contributes to the excitatory phase of peristalsis. To visualize the cholinergic and tachykinergic neuronal networks in our experimental settings, colon tissues were stained for Choline acetyltransferase (ChAT; enzyme responsible for the synthesis of acetylcholine, excitatory neurotransmitter), and for substance P, respectively [23]. A significant effect of microbiota on ChAT immunoreactivity was detected in the myenteric plexus (*P*_Microbiota_ = 0.0162), along with a sex‐dependent interaction (*P*_Interaction_ = 0.0008) (Figure 5A–C). Germ‐free female mice displayed an increased immunoreactivity for ChAT compared to males (*p* < 0.01; Figure 5A–C). In males, ChAT immunoreactivity was significantly increased in the SMM group compared with controls (*p* < 0.01; Figure 5A,C). In females, microbiota from the NSM and MFM groups significantly reduced ChAT immunofluorescence compared with controls (*p* < 0.01, Figure 5B,C). Furthermore, ChAT immunoreactivity in the SMM and MFM groups was significantly higher in males than in females (*p* < 0.05; Figure 5A–C). These findings suggest that the microbiota from infants fed with maternal secretor milk or dairy formula influenced the density of the myenteric cholinergic neuronal network with distinct effects observed between males and females, without affecting substance P‐immunoreactive nerve fibers (Figure 5D).

**FIGURE 5:**
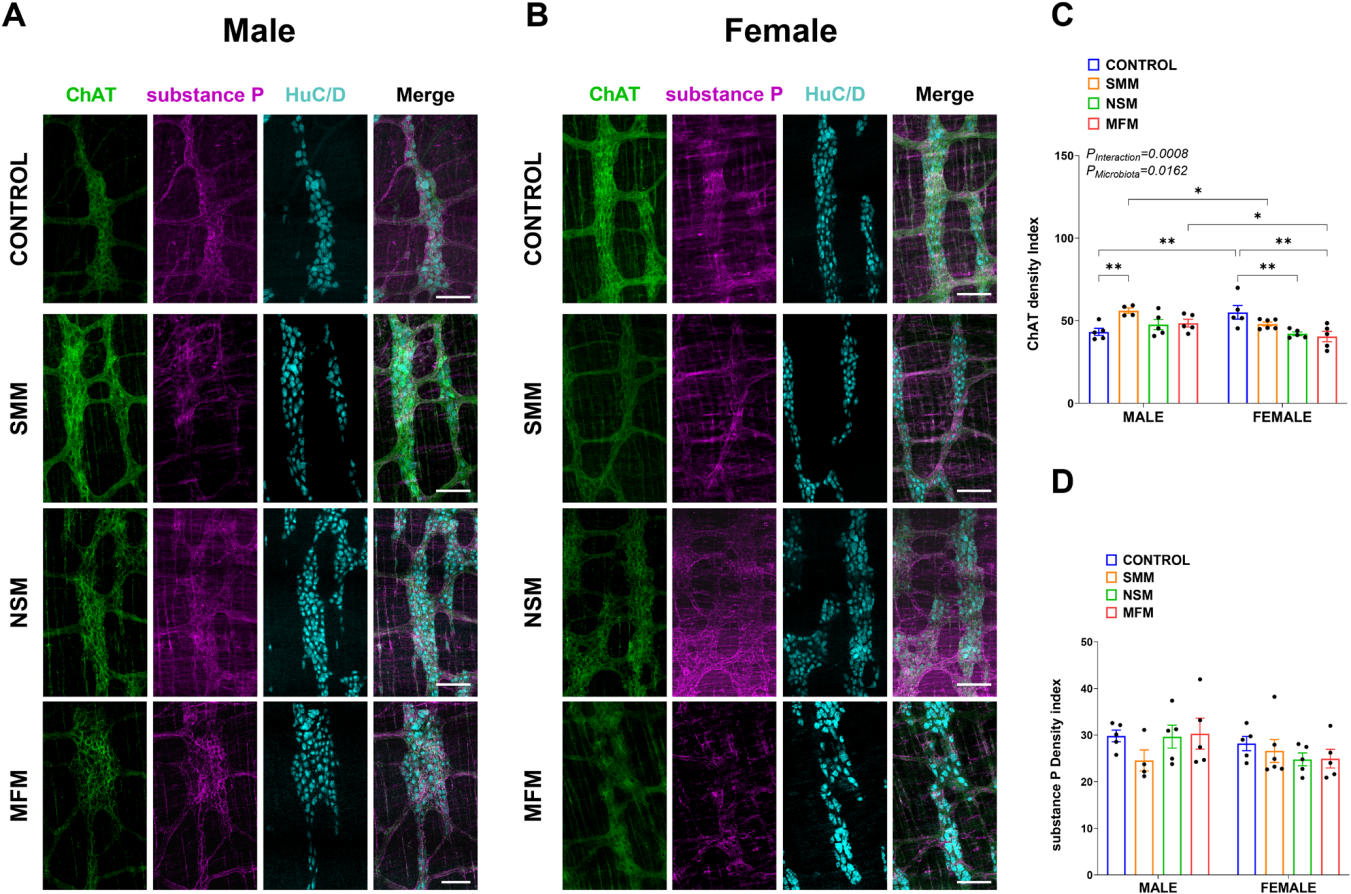
Enteric cholinergic neuronal network is affected by the infant microbiomes in a sex‐dependent manner. Representative confocal photomicrographs showing the distribution of ChAT (green, marker for cholinergic neurons), substance P (magenta; marker for tachykinergic neurons), and HuC/D (cyan; pan neuronal marker) in the myenteric plexus of male (A) and female (B) mouse colon. Scale bars = 100 μm. (C) ChAT and substance P (D) density indices in the myenteric plexus of male and female mice. Data are reported as mean ± SEM for panels (C) and (D). The effect of microbiota, sex, and their interactions was determined using a two‐way ANOVA with Tukey's multiple comparison test in GraphPad Prism Version 11 (www.graphpad.com). (C) **p < 0.01 male control vs. male SMM and female control; female control vs. female NSM and female MFM; *p < 0.05 male SMM vs. female SMM; *p < 0.05 male MFM vs. female MFM. SMM = mice transplanted with fecal material from infants consuming milk from secretor mothers, NSM = mice transplanted with fecal material from infants consuming milk from non‐secretor mothers, MFM = mice transplanted with fecal material from infants consuming dairy‐based formula, CONTROL = germ‐free mice. (*N* = 4–6 mice/sex/group).

### 3.6 A Two‐Week Microbiota Colonization of the Germ‐Free Mouse Intestinal Tract Does Not Influence the Development of Glial Cells in the Colon Myenteric Plexus

Considering the changes in the enteric neuronal network of germ‐free mice caused by microbiota, we sought to determine whether and to what extent the enteric glia was impacted by the introduction of microbes in the intestinal tract. For this purpose, colon tissues were labeled with the pan neuronal marker HuC/D and with glial markers GFAP and S100β, as previously described [24]. The proportion between glial cells and neurons per myenteric ganglion was calculated. Male germ‐free mice displayed a significantly higher number of GFAP^+^S100β^+^ cells per myenteric ganglia area compared to females, and this difference was maintained after microbiota transplantation (*P*_sex_ < 0.0001; Figure 6A–C). Accordingly, a two‐way ANOVA revealed an effect of sex (*P*_Sex_ < 0.0001) and the microbiota (*P*_Microbiota_ = 0.0482) on the ratio between glia and neurons, which was significantly higher in males compared to female mice (*p* < 0.01; Figure 6D). Glia‐to‐neuron ratio was still higher in males than females in the groups that received SMM and MFM microbiota (*p* < 0.01; Figure 6D). Moreover, the glia‐to‐neuron ratio of male SMM was significantly higher than that of sex‐matched NSM (*p* < 0.05). These findings indicate a higher glia‐to‐neuron ratio in juvenile germ‐free male mice compared to females, and these relationships seem to be not influenced by the presence of the microbiome.

**FIGURE 6:**
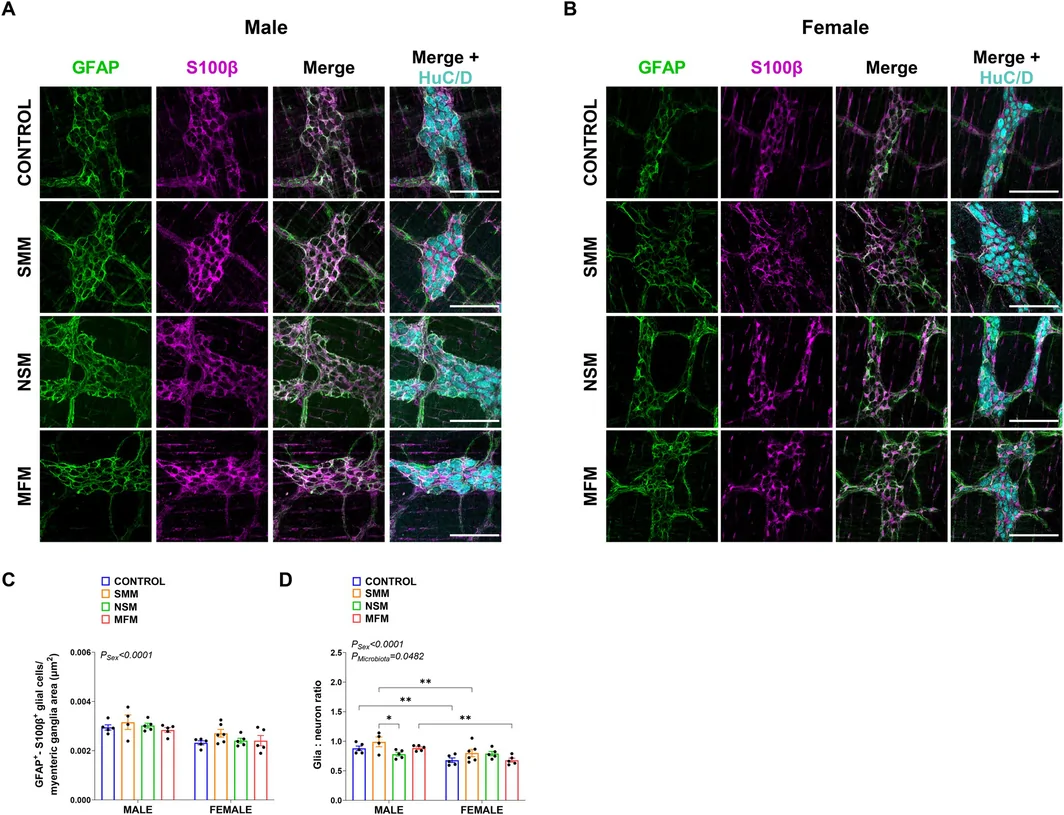
Infant microbiomes do not affect glial cell development in the myenteric plexus of juvenile mice. Representative confocal microphotographs showing the distribution of glial fibrillary acidic protein (GFAP) + (green), and S100β + (magenta) glial cells, and HuC/D+ neurons (cyan on the merge panel; pan neuronal marker) in the myenteric plexus of male (A) and female (B) mouse colon. Scale bars = 100 μm. Quantification of the number of GFAP+S100β+ glial cells (C) and the ratio of glia (GFAP+S100β+) to neurons (HuC/D+) (D) in myenteric ganglia of male and female mice. The effect of microbiota, sex and their interactions were determined using a two‐way ANOVA with Tukey's multiple comparison test in GraphPad Prism Version 11 (www.graphpad.com). (D) **p < 0.01 male control vs. female control; male SMM vs. female SMM; male MFM vs. female MFM; *p* < 0.05 male SMM vs. male NSM. SMM = mice transplanted with fecal material from infants consuming milk from secretor mothers, NSM = mice transplanted with fecal material from infants consuming milk from non‐secretor mothers, MFM = mice transplanted with fecal material from infants consuming dairy‐based formula, CONTROL = germ‐free mice. (*N* = 4–6 mice/sex/group).

## 4 Discussion

Early life is a critical period during which the gut microbiome and the ENS develop in parallel, establishing the foundations for long‐term GI function [3]. Furthermore, initial microbial colonization influences ENS maturation in various aspects, including neuronal differentiation, glial development, synaptic organization, and the physiological development of neuronally mediated intestinal motility.

In agreement with our previous results, infant feeding status (SMM or NSM) resulted in distinct clustering in mouse fecal microbiome composition. Fecal material transfer was performed at PND 21, a developmental stage critical for ENS maturation, during which the myenteric and submucosal plexuses are fully established but continue to undergo neuronal refinement, synaptogenesis, and enteric glial expansion. Furthermore, our experimental time‐point has solid translational relevance because the neonatal murine GI tract is developmentally less mature than that of humans [3]. Therefore, the post‐weaning period in mice provides an appropriate window to model microbiota‐driven ENS changes associated with human breastfeeding [3]. Notably, previous studies have demonstrated that microbial engraftment is significantly more effective when FMT is performed at weaning rather than in adulthood [19], further supporting our choice of intervention timing.

Our results suggest sex‐dependent alterations in ENS developmental outcomes, particularly in female mice. These findings are consistent with previous evidence suggesting that females exhibit heightened sensitivity to early‐life microbiota perturbations than males [19, 25], highlighting the importance of including the sex factor when investigating the impact of gut microbiome on ENS development. The results of this study suggest sex‐dependent patterns in GI motility that appear to be modulated by microbial colonization. In particular, female SMM mice exhibited a significant increase in distal GI transit efficiency compared with sex‐matched germ‐free controls after 180 min of fluorescent marker gavage, with no detectable changes in the male groups.

Several mechanisms might underlie this selective responsiveness in females, including differences in mucosal glycan expression, hormonal regulation of epithelial and ENS signaling, and immune–microbiota interactions that are known to vary between sexes [26]. Sex differences in vagal signaling, stress responses, and hypothalamic–pituitary–adrenal axis activity can influence both gut motility and ENS function. Estrogen‐mediated regulation of enteric neuronal development and function, together with sex‐specific host‐microbiota interactions, may render females more responsive to early‐life microbial perturbations and increase their susceptibility to functional disorders of gut‐brain interactions [27]. Additionally, sex differences in neuroimmune and neuroendocrine signaling pathways could contribute to the distinct ENS and motility phenotypes observed between males and females [28]. Secretor‐derived microbial communities are enriched for taxa capable of utilizing host fucosylated glycans, and females may provide a more permissive or metabolically supportive environment for these organisms to exert functional effects on gut transit efficiency [25]. Taken together, the evidence suggests a possible interaction between host sex and maternal secretor microbiota, with females exhibiting more pronounced responses to gut–brain axis perturbations and microbial‐dependent modulation of intestinal motility pathways than males.

The increased distal GI transit efficiency observed in female SMM aligns with the higher proportion of nitrergic neurons in colon myenteric ganglia compared to sex‐matched control and to male SMM. Early‐life antibiotic exposure has consistently been shown to disrupt the maturation of the ENS, particularly by reducing the population of nitrergic neurons that are essential for modulating the relaxation of the smooth musculature, thereby supporting normal intestinal motility. This neuronal loss is usually accompanied by delayed transit and constipation‐like phenotypes, as demonstrated across multiple models [11, 13, 29]. A possible explanation emerging from these studies is that early microbial depletion interrupts critical microbe–ENS signaling pathways that guide neuronal differentiation and maintenance. In particular, microbial products that are recognized by Toll‐like receptors (TLRs) appear to play a central role in supporting nitrergic neuron development. Prior research has shown that TLR2 and TLR4 signaling promotes enteric neurogenesis and protects against neuronal loss in the small intestine and in the colon [22, 30, 31, 32]. *Bacteroides* species are well‐known activators of TLR2, and this pathway has been implicated in supporting enteric neuronal populations and neural‐mediated colonic motility in adult germ‐free mice [33]. In our study, both female SMM and MFM groups exhibited a higher abundance of *Bacteroides* spp., raising the possibility that the recovery of nitrergic neurons observed in these groups may be driven by a *Bacteroides*‐mediated TLR2 signaling pathway.

Since motility patterns are regulated by the combined action of excitatory and inhibitory neuronal inputs, we assessed the principal components of excitatory neurotransmissions in the myenteric plexus, namely ChAT and substance P expressing excitatory neurons [33]. Our findings suggest a sex‐dependent regulation of excitatory cholinergic neurons in the myenteric plexus by gut microbes. In germ‐free conditions, females exhibited markedly higher ChAT immunoreactivity than males, suggesting that the absence of microbial signals disproportionately enhances excitatory cholinergic tone in the female ENS. This aligns with previous work showing that antibiotic‐induced microbiota disruptions in early life cause exaggerated neurochemical phenotypes and altered motility patterns due to disrupted excitatory–inhibitory balance with different patterns between females and males [13, 34]. Microbiota transplantation produced divergent effects between sexes: in males, ChAT immunoreactivity increased following SMM and MFM colonization, indicating that microbial communities help restore excitatory neurotransmissions. In contrast, females showed significant reductions in ChAT immunoreactivity in NSM and MFM groups, suggesting that microbial colonization normalizes an otherwise elevated cholinergic tone. These findings point toward a role for host sex in shaping how the microbiota modulate enteric cholinergic pathways during early ENS development.

Early‐life microbial colonization plays a critical role in shaping enteric glia development alongside neuronal maturation. Previous research has demonstrated that microbial signaling is essential for optimal glial maturation across the gut wall [9] and within the myenteric plexus [19]. Postnatally, enteric glial cells arise from progenitor populations in the myenteric plexus and continue to be replaced throughout adulthood. Both the development and ongoing renewal of murine enteric glial cells have been shown to depend on the microbiome, potentially through TLRs‐mediated pathways [35]. In our study, myenteric intraganglionic glia expressing GFAP and S100β, corresponding to enteric glia Type I [24], were more abundant in males than females. Likewise, the glia‐to‐neuron ratio was elevated in all male groups except the NSM cohort, suggesting that, within the limits of our sample size, infant‐derived microbiomes did not markedly alter myenteric glial density. Instead, sex‐dependent differences were more prominent than microbiome‐specific effects. Taken together, these preliminary findings support the idea that early microbial colonization may shape enteric neuronal development in a sex‐specific manner. Larger cohorts and mechanistic studies will be necessary to determine how microbial stimuli intersect with sex‐linked factors to influence enteric glia maturation and gut function.

Overall, our study findings support the idea that microbial colonization programmed by maternal secretor phenotype may influence intestinal motility and ENS development in a sex‐specific manner, warranting further investigation with larger cohorts.

### 4.1 Limitations and Future Directions

Several limitations should be considered when interpreting the findings from our study. First, the study did not include a comparison with conventionally raised mice, which could have provided important context for evaluating how early‐life microbial exposures differ from typical postnatal colonization. Second, the supplementation with HMO to sustain the growth of secretor‐derived microbiota could have improved our ability to fully assess how maternal secretor phenotype shapes microbial seeding and ENS development. As a future direction, it will be valuable to examine whether microbiota‐derived changes in gut motility persist into adulthood, particularly by combining early‐life microbial exposures with HMO supplementation to better mimic physiological conditions. Additionally, assessing the metabolic profile of the different microbial communities could help identify specific metabolites or pathways that mechanistically link the gut microbiome to ENS maturation. Together, this emerging body of work provides a conceptual framework for understanding how early‐life microbial environments shaped by maternal secretor status may influence ENS development, setting the basis for future translational investigations aimed at determining whether similar mechanisms occur in humans.

## Author Contributions

Bharath K. Kumar, Valentina Caputi, and Laxmi Yeruva: substantial contributions to the conception of the work, acquisition, analysis, and interpretation of data. Kirandeep K. Gill, Bindu Nanduri, Tanya Leroith, Renee Fox, Fernanda Rosa, and Joshua M. Lyte: substantial contributions to acquisition, analysis, and interpretation of data. Bharath K. Kumar and Valentina Caputi prepared the initial draft. Laxmi Yeruva, Kirandeep K. Gill, Bindu Nanduri, Tanya Leroith, Renee Fox, Fernanda Rosa, and Joshua M. Lyte revised the original draft critically for important intellectual content. All authors approved the final version of the manuscript to be published and agreed to be accountable for all aspects of the work.

## Funding

This work was supported, by the U.S. Department of Agriculture 6026‐10700‐001‐000D and 6026‐51000‐012‐000D. Dr. Aline Andres supported the IRB protocol to help collect the fecal samples from Happy Tummies study.

## Conflicts of Interest

The authors declare no conflicts of interest.

## Supporting information


**Figure S1:** Infant feeding status is associated with fecal microbiome composition in males. (A) Dissimilarity in gut microbiome composition among infant feeding groups as shown by Bray–Curtis β‐diversity. Group differences in β‐diversity were assessed using permutational multivariate analysis of variance (PERMANOVA) (I), and within‐group variation was evaluated using PERMDISP (II). (B) Differentially abundant bacterial genera among mice gavaged with SMM, NSM, and MFM, identified using linear discriminant analysis effect size (LEfSe; FDR5 < 0.05). (C) α‐diversity as shown by Chao1 (I), Shannon (II), and Simpson (III) indices. β‐diversity, α‐diversity, and LEfSe analysis were performed in MicrobiomeAnalyst (2.0). SMM = mice transplanted with fecal material from infants consuming milk from secretor mothers, NSM = mice transplanted with fecal material from infants consuming milk from non‐secretor mothers, MFM = mice transplanted with fecal material from infants consuming dairy‐based formula (*N* = 10–12 mice/group). *p ≤ 0.05, **p ≤ 0.01, ***p ≤ 0.001, ****p ≤ 0.0001.

**Figure S2:** Infant feeding status is associated with fecal microbiome composition in females. (A) Dissimilarity in gut microbiome composition among infant feeding groups as shown by Bray–Curtis β‐diversity. Group differences in β‐diversity were assessed using permutational multivariate analysis of variance (PERMANOVA) (I), and within‐group variation was evaluated using PERMDISP (II). (B) Differentially abundant bacterial genera among mice gavaged with SMM, NSM, and MFM, identified using linear discriminant analysis effect size (LEfSe; FDR5 < 0.05). (C) α‐diversity as shown by Chao1 (I), Shannon (II), and Simpson (III) indices. β‐diversity, α‐diversity, and LEfSe analysis were performed in MicrobiomeAnalyst (2.0). SMM = mice transplanted with fecal material from infants consuming milk from secretor mothers, NSM = mice transplanted with fecal material from infants consuming milk from non‐secretor mothers, MFM = mice transplanted with fecal material from infants consuming dairy‐based formula (*N* = 10–12 mice/group). *p ≤ 0.05, **p ≤ 0.01, ***p ≤ 0.001, ****p ≤ 0.0001.

**Figure S3:** Influence of infant feeding on gut morphology. Mice gavaged with fecal material from infants fed with either secretory or non‐secretory or milk formula did not show any differences in small intestine crypt depth or villi height (A and B—small intestine Segment 1 (SI1) crypt depth and villi height, respectively; C and D—small intestine Segment 5 (SI5) crypt depth and villi height, respectively) or colon and cecal gland depths (E and F, respectively). *p*‐Values were calculated using one‐way ANOVA with Tukey's multiple comparison tests in GraphPad Prism Version 11.0 (www.graphpad.com). SMM = mice transplanted with fecal material from infants consuming milk from secretor mothers, NSM = mice transplanted with fecal material from infants consuming milk from non‐secretor mothers, MFM = mice transplanted with fecal material from infants consuming dairy‐based formula, CONTROL = germ‐free mice (*N* = 4–8 mice/group).

**Table S1:** List of primary and secondary antibodies used in the immunohistochemistry experiments. Primary antibodies were diluted in blocking solution, while secondary antibodies were diluted in 10 mM PBS + 0.5% Triton‐X100.
**Table S2:** Tukey's multiple comparisons (GraphPad Prism Version 11; www.graphpad.com) between intestinal regions (within each group) of 250 kDa‐FITC dextran distribution in the GI tract of male mice after 40 min of oral administration.
**Table S3:** Tukey's multiple comparisons (GraphPad Prism Version 11; www.graphpad.com) between intestinal regions (within each group) of 250 kDa‐FITC dextran distribution in the GI tract of female mice after 40 min of oral administration.
**Table S4:** Tukey's multiple comparisons (GraphPad Prism Version 11; www.graphpad.com) between intestinal regions (within each group) of 250 kDa‐FITC dextran distribution in the GI tract of male mice after 180 min of oral administration.
**Table S5:** Tukey's multiple comparisons (GraphPad Prism Version 11; www.graphpad.com) between intestinal regions (within each group) of 250 kDa‐FITC dextran distribution in the GI tract of female mice after 180 min of oral administration.

## Data Availability

The data that support the findings of this study are available from the corresponding author upon reasonable request.
