# Supplementary material for: Microbiota From Human Infants Consuming Secretors Mothers' Milk Affects Murine Intestinal Motility and Enteric Nervous System Development in a Sex‐Dependent Manner
Source: Neurogastroenterol Motil. 2026 Nov 4;38(11):e70475. doi: 10.1111/nmo.70475 (PMC13732552; doi:10.1111/nmo.70475)
Supplement: Supplementary file 1 [file NMO-38-e70475-s001.pdf]

A

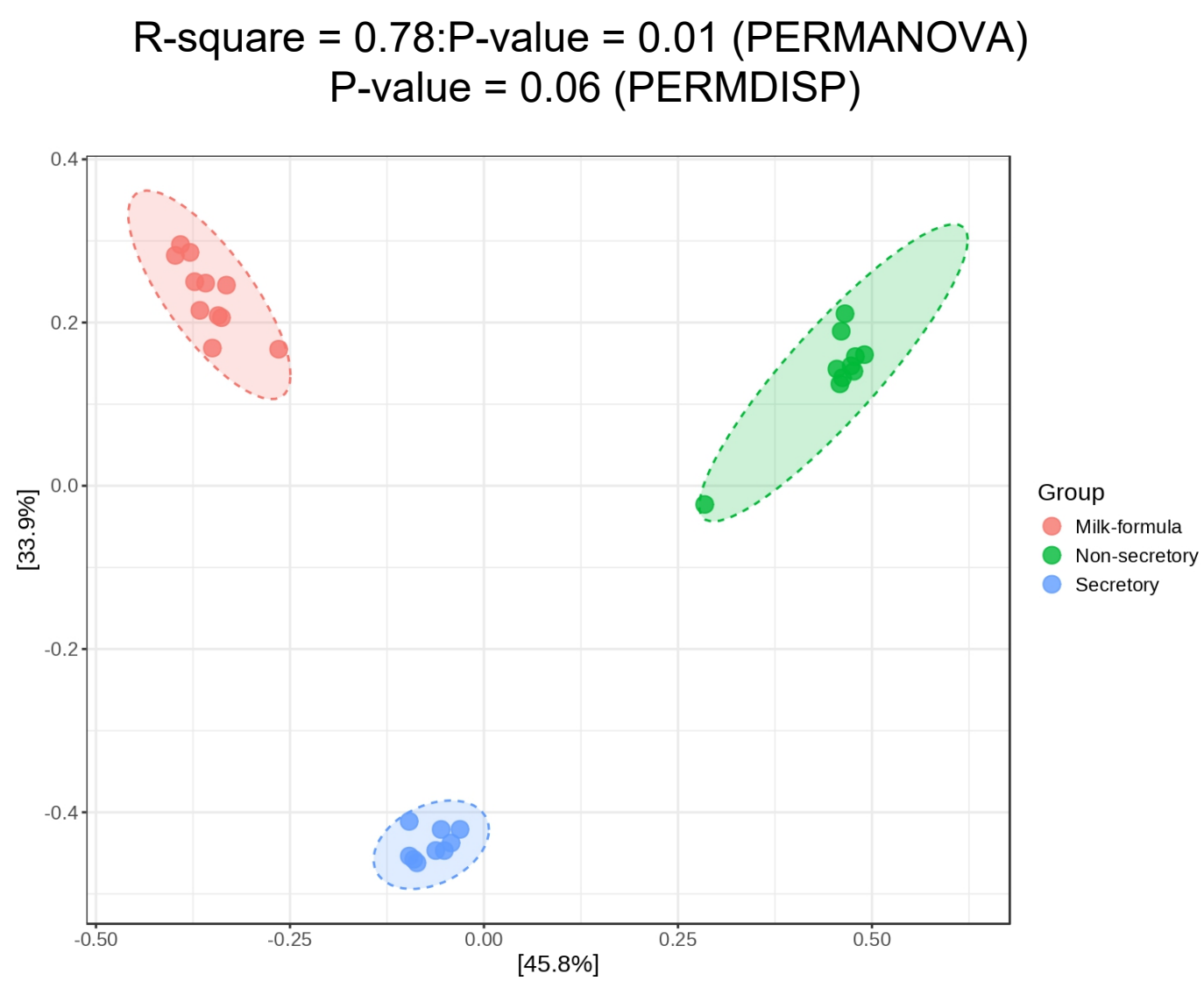

R-square = 0.78:P-value = 0.01 (PERMANOVA)
P-value = 0.06 (PERMDISP)
Group
Milk-formula
Non-secretory
Secretory
[45.8%]
[33.9%]

B

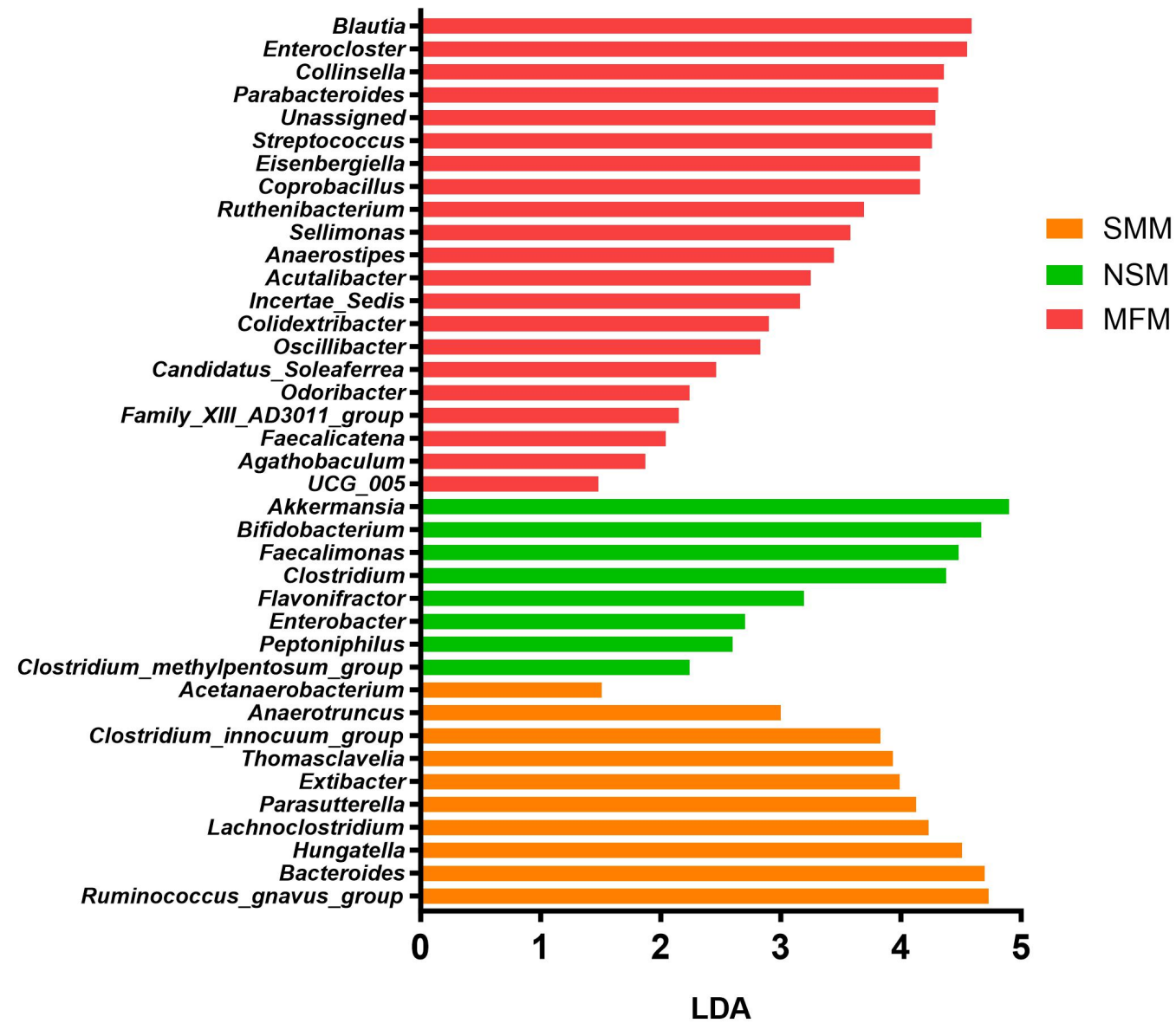

Blautia
Enterocloster
Collinsella
Parabacteroides
Unassigned
Streptococcus
Eisenbergiella
Coprobacillus
Ruthenibacterium
Sellimonas
Anaerostipes
Acutalibacter
Incertae_Sedis
Colidextribacter
Oscillibacter
Candidatus_Soleaferrea
Odoribacter
Family_XIII_AD3011_group
Faecalicatena
Agathobaculum
UCG_005
Akkermansia
Bifidobacterium
Faecalimonas
Clostridium
Flavonifractor
Enterobacter
Peptoniphilus
Clostridium_methylpentosum_group
Acetanaerobacterium
Anaerotruncus
Clostridium_innocuum_group
Thomasclavelia
Extibacter
Parasutterella
Lachnoclostridium
Hungatella
Bacteroides
Ruminococcus_gnavus_group
LDA
SMM
NSM
MFM

C

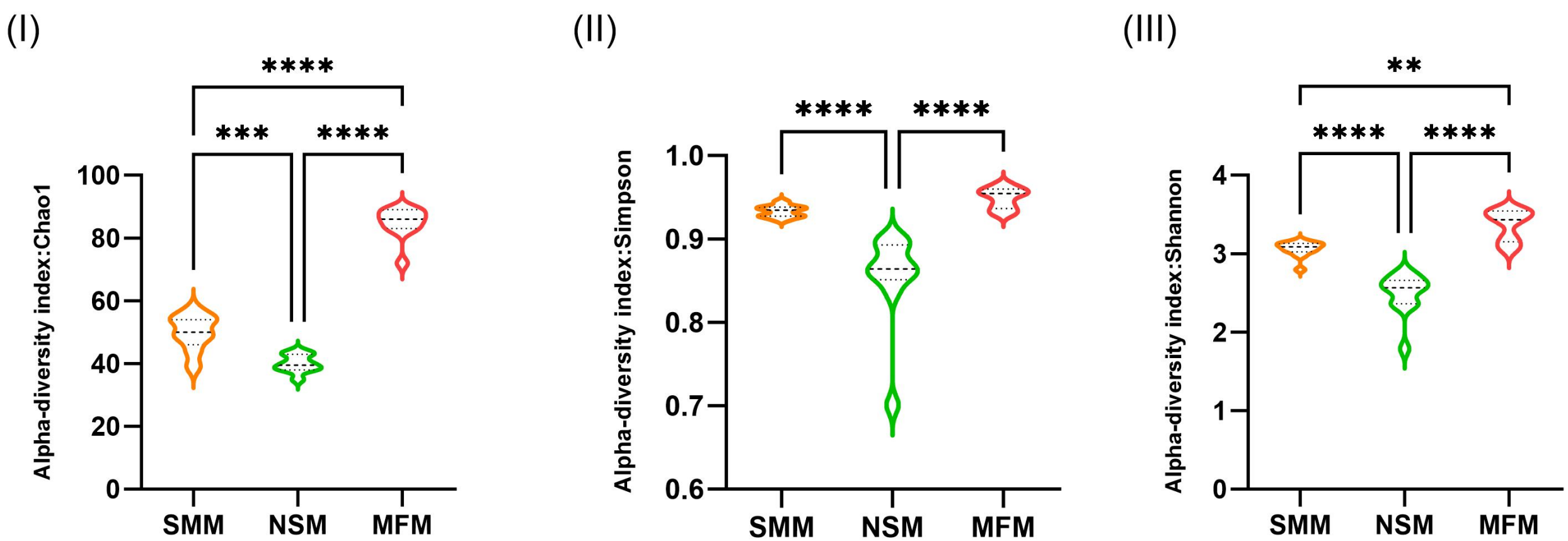

(I)
(II)
(III)
Alpha-diversity index:Chao1
Alpha-diversity index:Simpson
Alpha-diversity index:Shannon
SMM
NSM
MFM
