# Supplementary material for: Microbiota From Human Infants Consuming Secretors Mothers' Milk Affects Murine Intestinal Motility and Enteric Nervous System Development in a Sex‐Dependent Manner
Source: Neurogastroenterol Motil. 2026 Nov 4;38(11):e70475. doi: 10.1111/nmo.70475 (PMC13732552; doi:10.1111/nmo.70475)
Supplement: Supplementary file 2 [file NMO-38-e70475-s002.pdf]

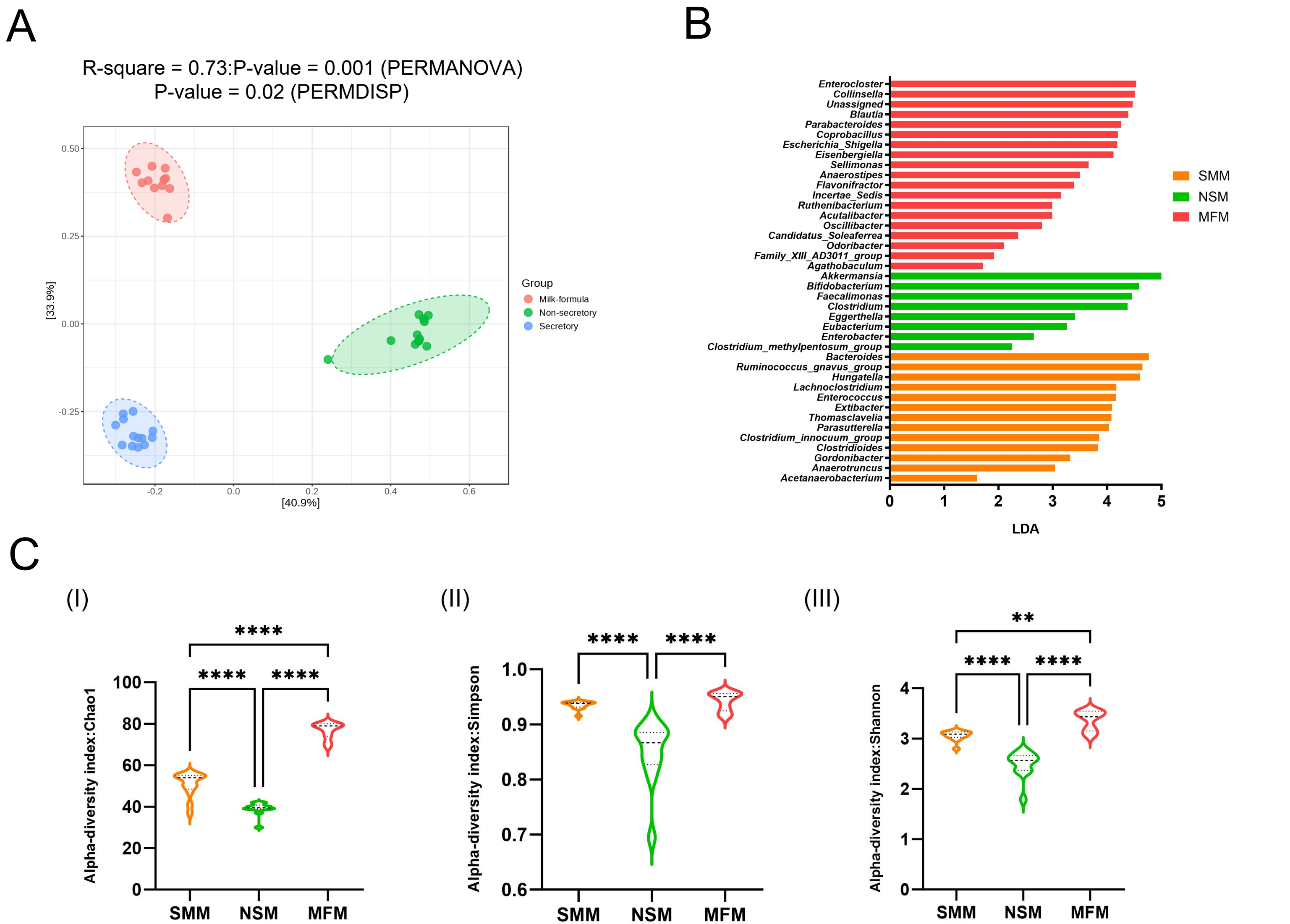
A
R-square = 0.73:P-value = 0.001 (PERMANOVA)
P-value = 0.02 (PERMDISP)
[33.9%]
[40.9%]
Group
Milk-formula
Non-secretory
Secretory
B
Enterocloster
Collinsella
Unassigned
Blautia
Parabacteroides
Coprobacillus
Escherichia_Shigella
Eisenbergiella
Sellimonas
Anaerostipes
Flavonifractor
Incertae_Sedis
Ruthenibacterium
Acutalibacter
Oscillibacter
Candidatus_Soleaferrea
Odoribacter
Family_XIII_AD3011_group
Agathobaculum
Akkermansia
Bifidobacterium
Faecalimonas
Clostridium
Eggerthella
Eubacterium
Enterobacter
Clostridium_methylpentosum_group
Bacteroides
Ruminococcus_gnavus_group
Hungatella
Lachnoclostridium
Enterococcus
Extibacter
Thomasclavelia
Parasutterella
Clostridium_innocuum_group
Clostridioides
Gordonibacter
Anaerotruncus
Acetanaerobacterium
LDA
SMM
NSM
MFM
C
(I)
Alpha-diversity index:Chao1
(II)
Alpha-diversity index:Simpson
(III)
Alpha-diversity index:Shannon
SMM NSM MFM
