# Supplementary material for: Microbiota From Human Infants Consuming Secretors Mothers' Milk Affects Murine Intestinal Motility and Enteric Nervous System Development in a Sex‐Dependent Manner
Source: Neurogastroenterol Motil. 2026 Nov 4;38(11):e70475. doi: 10.1111/nmo.70475 (PMC13732552; doi:10.1111/nmo.70475)
Supplement: Supplementary file 3 [file NMO-38-e70475-s003.pdf]

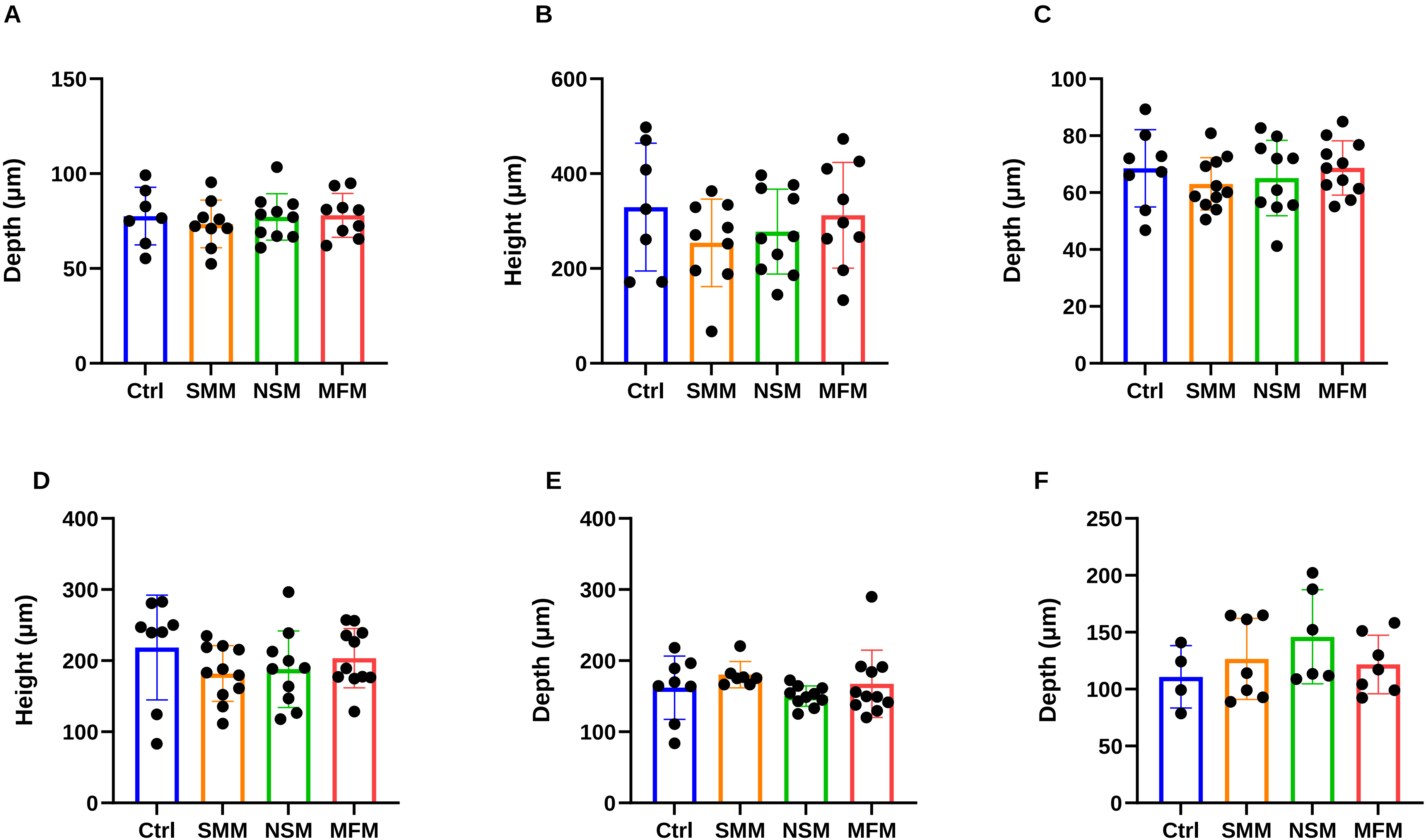
A
B
C
D
E
F
Depth (µm)
Height (µm)
Ctrl
SMM
NSM
MFM
0
50
100
150
200
250
300
400
600
20
40
60
80
