# Supplementary material for: Microbiota From Human Infants Consuming Secretors Mothers' Milk Affects Murine Intestinal Motility and Enteric Nervous System Development in a Sex‐Dependent Manner
Source: Neurogastroenterol Motil. 2026 Nov 4;38(11):e70475. doi: 10.1111/nmo.70475 (PMC13732552; doi:10.1111/nmo.70475)
Supplement: Supplementary file 4 [file NMO-38-e70475-s004.docx]

**SUPPORTING INFORMATION**

**METHODS**

**Preparation of intestinal full-thickness tissues for immunohistochemistry**

Full-thickness colon segments were prepared as previously described (Faggin et al., 2026). After removing the luminal contents (used for the GI motility experiments), colon segments were placed on a petri dish***.*** One end of the segment was sealed with a double knot using cotton thread. The segment was filled with 4 % paraformaldehyde (PFA) (in 10 mM PBS), flushed into the lumen from the open end with a 1ml button-needle syringe. The open end of the segment was sealed with cotton thread and the fixative-filled “sausage-like” segments were submerged in 4% PFA solution (20 ml/intestinal region) and placed on wet ice for 3 hours. Then, sausage-like segments were cut open at both ends and the fixative was washed away with 3 x 30 min washes with 10 mM PBS at room temperature (RT) and gentle shaking. Finally, segments were placed in 10 mM PBS + 0.05% (v/v) Thimerosal (cat# T5125, Millipore Sigma) and stored at 4 °C until use.

**Immunostaining and tissue clearing**

*Immunostaining*

Colon segments were cut into 1 cm-pieces and opened along the mesenteric border. Flat full-thickness tissues were placed in 10 mM PBS on a 48-well plate (untreated) and rinsed for 15 min with gentle shaking (RT). Tissues were then incubated for 20 hours in blocking solution (at RT) containing 5% Normal Horse Serum (cat#ab7484, Abcam), 5% Bovine Serum Albumin (cat#A7030, Millipore Sigma), 0.75% Triton X-100 (cat#T9284, Millipore Sigma) in 10 mM PBS. Double and triple labelling were performed during consecutive incubation times. Following overnight incubation at 4˚C with the first primary antibody (double labelling, **Supplementary Table 1**) or first two primary antibodies (triple labelling, **Supplementary Table 1**), preparations were washed 3 x 10 min with washing buffer containing 0.05% Triton X-100 and 0.5% 1-thioglycerol (cat#88640, Millipore Sigma) in 10 mM PBS. Intestinal preparations were then incubated for 20 hours at RT with the appropriate secondary antibody (double labelling) or antibodies (triple labelling) conjugated to a fluorophore (**Supplementary Table 1**). The second primary antibody (against HuC/D, **Supplementary Table 1**) was then incubated overnight at 4˚C and, after 3 x 10-min washes in washing buffer, the appropriate secondary antibody was added for 20 hours at RT. To obtain negative controls and ensure specificity of the immunostaining, a subset of colon tissues was stained with secondary antibodies in absence of primary antibodies. Tissues were then rinsed 5 x 30 minutes at RT with washing buffer and subjected to aqueous-based clearing procedure, as described below.

*Tissue clearing*

Stained tissues were cleared using the Ce3D procedure described by Bossolani et al. (Bossolani et al., 2019). After washing, colon tissues were dried on paper (Kim Tech wipes) and placed on Eppendorf tubes prefilled with Ce3D tissue clearing solution containing 20% N-methyl acetamide solution in 10 mM PBS, 80% (w/v) Histodenz, 0.1% Triton-X100 and 0.5% 1-thioglycerol until completely transparent (up to 12 hours at RT). Cleared stained tissues were kept in the Eppendorf tubes filled with Ce3D clarity solution and stored at RT (in the dark) until imaging.

**Confocal microscopy**

Stained tissues were imaged with a Leica Stellaris 5 laser scanning confocal microscope (Leica, Houston, TX, US). Each intestinal tissue, submerged in Ce3D clarity solution was positioned between two cover glasses #1.5 (24 x 50 mm), and the cover glasses were sealed with tape at both widths to prevent the tissue from floating during the imaging. Fluorophores were visualized using an OPSL 488 visible laser for Alexa Fluor 488, a DPSS 561 visible laser for Alexa Fluor 568, and a Diode 638 laser for Alexa Fluor 633. Imaging with each laser was performed sequentially using frame averaging. All microscope settings (lasers power, detector gain, pinhole) were set to collect images below saturation and were kept consistent for all images.

Colon tissues were imaged with HC PL APO CS2 10X/0.40 dry objective (working distance=2.56 mm; 3.0 zoom). Full-thickness Z-stacks were used for determining the areas of myenteric ganglia in each intestinal region and for assessing neuron subtype and glial cells counting. Considering the large spatial extension of myenteric neuronal structures within each intestinal region, we acquired multiple partial images and stitched them together to obtain the final complete image using the tile scan function of the Navigator tool. Complete tile scans (10% overlap) consisted of 20 to 40 images of 512 x 512-pixel resolution and 8-bit depth. Each Z-axis step for the images taken with the 10X objective was 2.4 μm. Two complete tile scans of 20-40 tiles (about 14 mm^2^) were acquired for each mouse/group.

**Image analysis**

All image analyses were performed in a blinded fashion using FIJI software (Fiji is just Image J, version 1.54p).

*Double labelling HuC/D-nNOS*

Myenteric ganglia areas were measured in 2D-maximum projections images by tracing boundaries around neuronal cellular bodies stained with HuC/D using the freehand tool of FIJI software. To determine whether agglomerates of neuronal cells as well as single cells were included or not in the boundaries of the ganglia, we first determined the average size of neuronal cells. Each neuronal cell area (μm^2^) was computed and used to calculate the maximum and minimum Feret diameters. Boundaries of myenteric ganglia were determined as locations where the distance of two agglomerates of cells was less than the *average Feret diameter* of one neuronal cell (Beebe et al., 2016). The analysis of the total neuron population in the myenteric ganglia was performed by counting HuC/D+ cells in 15-20 ganglia/image/mouse. The number of HuC/D+ neurons was normalized per myenteric ganglion area (µm^2^), as previously described (Faggin et al., 2025a). The proportion of nNOS+ neurons was established by double fluorescently labeling HuC/D+-nNOS+ myenteric neurons per ganglion (15-20 ganglia/image/mouse). The number of HuC/D+-nNOS+ myenteric neurons was normalized per myenteric ganglion area (µm^2^). The percentage of HuC/D+-nNOS+ myenteric neurons on the total HuC/D+ neurons was also calculated.

*Triple labelling HuC/D-ChAT-substance P*

Changes in Choline acetyltransferase (ChAT) and substance P (SP) immunoreactivities were assessed by and analyzing the density index of ChAT+ or substance P+ labeling for each myenteric ganglion area (µm^2^). Ganglionic boundaries were outlined by tracing around ChAT+-substance P+ nerve strands surrounding HuC/D+ cells in three-channels 2D-maximum projection images (8-10 ganglia/image/mouse), and each ganglion area was considered a ROI. Two-channels Z-stack 3D images were split into respective color channels (green-ChAT; magenta-substance P) and, after subtracting the background, each channel (as a Z-stack) was converted to binary using the triangle threshold setting of FIJI. Binary 3D-Z-stacks were then converted to 2D-maximum projection images, and the intensity of ChAT+ or substance P+ immunofluorescence was determined as area fraction limited to the staining threshold (i.e., density index) within each myenteric ganglion (ROI).

*Triple labelling HuC/D-GFAP-S100β*

Changes in enteric glia populations and glia-to-neuron ratio were assessed by counting GFAP+-S100β+ cell bodies (glia) and HuC/D+ cell bodies (neurons) in each myenteric ganglia area (8-10 ganglia/image/mouse), as previously described (Frith et al., 2024). Specifically, glial cells were defined as HuC/D-negative and GFAP+ S100β+ cells within the border of GFAP/S100β staining delineating the ganglion.

**SUPPLEMENTARY TABLES**

**Supplementary Table 1:** List of primary and secondary antibodies used in the immunohistochemistry experiments. Primary antibodies were diluted in blocking solution, while secondary antibodies were diluted in in 10 mM PBS + 0.5% Triton-X100.

| **Antibody** | **Host Species** | **Dilution** | **Catalog Number** | **Source** |
| --- | --- | --- | --- | --- |
| ***Primary Antibody***  ***(Clone)*** |  |  |  |  |
| HuC/D  (monoclonal) | Mouse biotin-conjugated | 1:100 | A-21272 | ThermoFisher Scientific |
| nNOS  (polyclonal) | Rabbit | 1:100 | 61-700 | ThermoFisher Scientific |
| GFAP  (polyclonal) | Chicken | 1:100 | ab4674 | Abcam |
| S100β  (EP1576Y) | Rabbit | 1:100 | ab52642 | Abcam |
| ChAT  (polyclonal) | Goat | 1:50 | AB144P | Millipore  Sigma-Aldrich |
| Substance P  (polyclonal) | rabbit | 1:100 | 20064 | Immunostar |
| ***Secondary Antibody*** |  |  |  |  |
| Donkey anti-Rabbit IgG (H+L) Highly Cross-Adsorbed Secondary Antibody, Alexa Fluor™ 568 | Donkey | 1:300 | A10042 | ThermoFisher Scientific |
| Donkey anti-Goat IgG (H+L) Cross-Adsorbed Secondary Antibody, Alexa Fluor™ 488 | Donkey | 1:300 | A-11055 | ThermoFisher Scientific |
| Anti-Chicken IgY (H+L), highly cross-adsorbed, CF™ 488A antibody produced in donkey | Donkey | 1:300 | SAB4600031 | Millipore  Sigma-Aldrich |
| Streptavidin, Alexa Fluor™ 633 conjugate | N/A | 1:300 | S-21375 | ThermoFisher Scientific |

**Supplementary Table 2:** Tukey`s multiple comparisons (GraphPad Prism Version 11; [www.graphpad.com](http://www.graphpad.com)) between intestinal regions (within each group) of 250 kDa-FITC dextran distribution in the GI tract of male mice after 40 min of oral administration.

| **MALE**  **(Exp 40 min)** | **CONTROL** | | **SMM** | | **NSM** | | **MFM** | |
| --- | --- | --- | --- | --- | --- | --- | --- | --- |
| Intestinal regions |  | P  -VALUE |  | P-VALUE |  | P-VALUE |  | P-VALUE |
| Sto vs. SI 1 | ns | >0.9999 | ns | >0.9999 | ns | >0.9999 | ns | >0.9999 |
| Sto vs. SI 2 | ns | >0.9999 | ns | >0.9999 | ns | >0.9999 | ns | >0.9999 |
| Sto vs. SI 3 | ns | 0.9798 | ns | 0.5644 | * | 0.0493 | ns | 0.1756 |
| Sto vs. SI 4 | ** | 0.0023 | ** | 0.0021 | *** | 0.0004 | **** | <0.0001 |
| Sto vs. SI 5 | ns | 0.9695 | ns | 0.2139 | ns | 0.6158 | ns | 0.9984 |
| Sto vs. SI 6 | ns | 0.9999 | ns | >0.9999 | ns | >0.9999 | ns | >0.9999 |
| Sto vs. Cecum | ns | >0.9999 | ns | >0.9999 | ns | >0.9999 | ns | >0.9999 |
| Sto vs. Colon | ns | >0.9999 | ns | >0.9999 | ns | >0.9999 | ns | >0.9999 |
| SI 1 vs. SI 2 | ns | >0.9999 | ns | >0.9999 | ns | >0.9999 | ns | 0.9995 |
| SI 1 vs. SI 3 | ns | 0.9962 | ns | 0.7668 | * | 0.0461 | ns | 0.1217 |
| SI 1 vs. SI 4 | ** | 0.0054 | ** | 0.0063 | *** | 0.0004 | **** | <0.0001 |
| SI 1 vs. SI 5 | ns | 0.9932 | ns | 0.3801 | ns | 0.5997 | ns | 0.9937 |
| SI 1 vs. SI 6 | ns | 0.9979 | ns | >0.9999 | ns | >0.9999 | ns | >0.9999 |
| SI 1 vs. Cecum | ns | 0.9994 | ns | >0.9999 | ns | >0.9999 | ns | >0.9999 |
| SI 1 vs. Colon | ns | 0.9989 | ns | >0.9999 | ns | >0.9999 | ns | >0.9999 |
| SI 2 vs. SI 3 | ns | 0.9908 | ns | 0.8105 | ns | 0.0702 | ns | 0.4201 |
| SI 2 vs. SI 4 | ** | 0.0036 | ** | 0.0083 | *** | 0.0007 | *** | 0.0003 |
| SI 2 vs. SI 5 | ns | 0.9851 | ns | 0.4298 | ns | 0.7018 | ns | >0.9999 |
| SI 2 vs. SI 6 | ns | 0.9994 | ns | >0.9999 | ns | >0.9999 | ns | 0.9995 |
| SI 2 vs. Cecum | ns | 0.9999 | ns | >0.9999 | ns | >0.9999 | ns | 0.9997 |
| SI 2 vs. Colon | ns | 0.9997 | ns | >0.9999 | ns | >0.9999 | ns | 0.9997 |
| SI 3 vs. SI 4 | ns | 0.069 | ns | 0.4501 | ns | 0.9073 | ns | 0.2644 |
| SI 3 vs. SI 5 | ns | >0.9999 | ns | 0.9997 | ns | 0.9421 | ns | 0.5948 |
| SI 3 vs. SI 6 | ns | 0.821 | ns | 0.693 | * | 0.0383 | ns | 0.1254 |
| SI 3 vs. Cecum | ns | 0.8769 | ns | 0.5536 | * | 0.0487 | ns | 0.1337 |
| SI 3 vs. Colon | ns | 0.8531 | ns | 0.5365 | * | 0.0463 | ns | 0.1367 |
| SI 4 vs. SI 5 | ns | 0.0836 | ns | 0.8266 | ns | 0.1852 | *** | 0.0007 |
| SI 4 vs. SI 6 | *** | 0.0003 | ** | 0.0042 | *** | 0.0003 | **** | <0.0001 |
| SI 4 vs. Cecum | *** | 0.0005 | ** | 0.002 | *** | 0.0004 | **** | <0.0001 |
| SI 4 vs. Colon | *** | 0.0004 | ** | 0.0018 | *** | 0.0004 | **** | <0.0001 |
| SI 5 vs. SI 6 | ns | 0.7814 | ns | 0.3096 | ns | 0.5554 | ns | 0.9943 |
| SI 5 vs. Cecum | ns | 0.844 | ns | 0.2071 | ns | 0.6127 | ns | 0.9954 |
| SI 5 vs. Colon | ns | 0.8171 | ns | 0.1965 | ns | 0.6008 | ns | 0.9957 |
| SI 6 vs. Cecum | ns | >0.9999 | ns | >0.9999 | ns | >0.9999 | ns | >0.9999 |
| SI 6 vs. Colon | ns | >0.9999 | ns | >0.9999 | ns | >0.9999 | ns | >0.9999 |
| Cecum vs. Colon | ns | >0.9999 | ns | >0.9999 | ns | >0.9999 | ns | >0.9999 |

**Supplementary Table 3:** Tukey`s multiple comparisons (GraphPad Prism Version 11; [www.graphpad.com](http://www.graphpad.com)) between intestinal regions (within each group) of 250 kDa-FITC dextran distribution in the GI tract of female mice after 40 min of oral administration.

| **FEMALE**  **(Exp 40 min)** | **CONTROL** | | **SMM** | | **NSM** | | **MFM** | |
| --- | --- | --- | --- | --- | --- | --- | --- | --- |
| Intestinal regions |  | P-VALUE |  | P-VALUE |  | P-VALUE |  | P-VALUE |
| Sto vs. SI 1 | ns | >0.9999 | ns | >0.9999 | ns | >0.9999 | ns | >0.9999 |
| Sto vs. SI 2 | ns | >0.9999 | ns | 0.9929 | ns | >0.9999 | ns | 0.9258 |
| Sto vs. SI 3 | ns | 0.999 | ns | 0.5433 | ns | 0.9915 | ns | >0.9999 |
| Sto vs. SI 4 | *** | 0.0004 | ** | 0.002 | **** | <0.0001 | ** | 0.0011 |
| Sto vs. SI 5 | ns | 0.0671 | ns | >0.9999 | ns | 0.6399 | ns | >0.9999 |
| Sto vs. SI 6 | ns | >0.9999 | ns | >0.9999 | ns | >0.9999 | ns | 0.9996 |
| Sto vs. Cecum | ns | >0.9999 | ns | >0.9999 | ns | >0.9999 | ns | 0.9998 |
| Sto vs. Colon | ns | >0.9999 | ns | >0.9999 | ns | >0.9999 | ns | 0.9998 |
| SI 1 vs. SI 2 | ns | >0.9999 | ns | 0.9973 | ns | >0.9999 | ns | 0.8559 |
| SI 1 vs. SI 3 | ns | >0.9999 | ns | 0.6242 | ns | 0.9872 | ns | 0.9998 |
| SI 1 vs. SI 4 | ** | 0.0013 | ** | 0.0031 | **** | <0.0001 | *** | 0.0005 |
| SI 1 vs. SI 5 | ns | 0.1482 | ns | >0.9999 | ns | 0.5996 | ns | >0.9999 |
| SI 1 vs. SI 6 | ns | >0.9999 | ns | >0.9999 | ns | >0.9999 | ns | >0.9999 |
| SI 1 vs. Cecum | ns | >0.9999 | ns | >0.9999 | ns | >0.9999 | ns | >0.9999 |
| SI 1 vs. Colon | ns | >0.9999 | ns | >0.9999 | ns | >0.9999 | ns | >0.9999 |
| SI 2 vs. SI 3 | ns | >0.9999 | ns | 0.9721 | ns | 0.9937 | ns | 0.9887 |
| SI 2 vs. SI 4 | ** | 0.0014 | * | 0.0399 | **** | <0.0001 | ns | 0.0785 |
| SI 2 vs. SI 5 | ns | 0.1541 | ns | 0.9909 | ns | 0.6672 | ns | 0.9752 |
| SI 2 vs. SI 6 | ns | >0.9999 | ns | 0.9802 | ns | >0.9999 | ns | 0.6071 |
| SI 2 vs. Cecum | ns | >0.9999 | ns | 0.9878 | ns | >0.9999 | ns | 0.6433 |
| SI 2 vs. Colon | ns | >0.9999 | ns | 0.9861 | ns | >0.9999 | ns | 0.6392 |
| SI 3 vs. SI 4 | ** | 0.0047 | ns | 0.4617 | **** | <0.0001 | ** | 0.0039 |
| SI 3 vs. SI 5 | ns | 0.3074 | ns | 0.5205 | ns | 0.99 | ns | >0.9999 |
| SI 3 vs. SI 6 | ns | 0.9973 | ns | 0.4402 | ns | 0.9868 | ns | 0.9894 |
| SI 3 vs. Cecum | ns | 0.9981 | ns | 0.4914 | ns | 0.9893 | ns | 0.9928 |
| SI 3 vs. Colon | ns | 0.9978 | ns | 0.4778 | ns | 0.9855 | ns | 0.9924 |
| SI 4 vs. SI 5 | ns | 0.8421 | ** | 0.0018 | ** | 0.0021 | ** | 0.0025 |
| SI 4 vs. SI 6 | *** | 0.0002 | ** | 0.0011 | **** | <0.0001 | **** | <0.0001 |
| SI 4 vs. Cecum | *** | 0.0003 | ** | 0.0015 | **** | <0.0001 | *** | 0.0001 |
| SI 4 vs. Colon | *** | 0.0003 | ** | 0.0014 | **** | <0.0001 | *** | 0.0001 |
| SI 5 vs. SI 6 | ns | 0.0502 | ns | >0.9999 | ns | 0.5965 | ns | 0.996 |
| SI 5 vs. Cecum | ns | 0.0557 | ns | >0.9999 | ns | 0.6185 | ns | 0.9975 |
| SI 5 vs. Colon | ns | 0.0533 | ns | >0.9999 | ns | 0.5871 | ns | 0.9973 |
| SI 6 vs. Cecum | ns | >0.9999 | ns | >0.9999 | ns | >0.9999 | ns | >0.9999 |
| SI 6 vs. Colon | ns | >0.9999 | ns | >0.9999 | ns | >0.9999 | ns | >0.9999 |
| Cecum vs. Colon | ns | >0.9999 | ns | >0.9999 | ns | >0.9999 | ns | >0.9999 |

**Supplementary Table 4:** Tukey`s multiple comparisons (GraphPad Prism Version 11; [www.graphpad.com](http://www.graphpad.com)) between intestinal regions (within each group) of 250 kDa-FITC dextran distribution in the GI tract of male mice after 180 min of oral administration.

| **MALE**  **(Exp 180 min)** | **CONTROL** | | **SMM** | | **NSM** | | **MFM** | |
| --- | --- | --- | --- | --- | --- | --- | --- | --- |
| Intestinal regions |  | P-VALUE |  | P-VALUE |  | P-VALUE |  | P-VALUE |
| Sto vs. SI 1 | ns | >0.9999 | ns | >0.9999 | ns | >0.9999 | ns | 0.9973 |
| Sto vs. SI 2 | ns | >0.9999 | ns | >0.9999 | ns | >0.9999 | ns | >0.9999 |
| Sto vs. SI 3 | ns | >0.9999 | ns | >0.9999 | ns | >0.9999 | ns | >0.9999 |
| Sto vs. SI 4 | ns | >0.9999 | ns | >0.9999 | ns | >0.9999 | ns | >0.9999 |
| Sto vs. SI 5 | ns | 0.4553 | ns | >0.9999 | ns | >0.9999 | ns | >0.9999 |
| Sto vs. SI 6 | ns | >0.9999 | ns | 0.9995 | ns | 0.8584 | ns | 0.9922 |
| Sto vs. Cecum | *** | 0.0006 | **** | <0.0001 | *** | 0.0001 | **** | <0.0001 |
| Sto vs. Colon 1 | ns | 0.1636 | ns | 0.8906 | ns | >0.9999 | * | 0.0198 |
| Sto vs. Colon 2 | ns | 0.8519 | ns | 0.6245 | ns | 0.9599 | ns | 0.3874 |
| Sto vs. Colon 3 | ns | >0.9999 | ns | 0.8729 | ns | 0.9852 | ns | >0.9999 |
| SI 1 vs. SI 2 | ns | >0.9999 | ns | 0.9999 | ns | >0.9999 | ns | 0.9994 |
| SI 1 vs. SI 3 | ns | >0.9999 | ns | >0.9999 | ns | >0.9999 | ns | 0.9967 |
| SI 1 vs. SI 4 | ns | >0.9999 | ns | >0.9999 | ns | >0.9999 | ns | 0.9982 |
| SI 1 vs. SI 5 | ns | 0.4187 | ns | >0.9999 | ns | 0.9979 | ns | 0.9999 |
| SI 1 vs. SI 6 | ns | >0.9999 | ns | 0.9993 | ns | 0.5408 | ns | >0.9999 |
| SI 1 vs. Cecum | *** | 0.0005 | **** | <0.0001 | **** | <0.0001 | **** | <0.0001 |
| SI 1 vs. Colon 1 | ns | 0.1441 | ns | 0.8797 | ns | 0.9909 | ns | 0.2425 |
| SI 1 vs. Colon 2 | ns | 0.8248 | ns | 0.6058 | ns | 0.7507 | ns | 0.9365 |
| SI 1 vs. Colon 3 | ns | >0.9999 | ns | 0.861 | ns | 0.8482 | ns | 0.9988 |
| SI 2 vs. SI 3 | ns | >0.9999 | ns | >0.9999 | ns | >0.9999 | ns | >0.9999 |
| SI 2 vs. SI 4 | ns | >0.9999 | ns | >0.9999 | ns | >0.9999 | ns | >0.9999 |
| SI 2 vs. SI 5 | ns | 0.4395 | ns | >0.9999 | ns | >0.9999 | ns | >0.9999 |
| SI 2 vs. SI 6 | ns | >0.9999 | ns | >0.9999 | ns | 0.7523 | ns | 0.9977 |
| SI 2 vs. Cecum | *** | 0.0005 | **** | <0.0001 | **** | <0.0001 | **** | <0.0001 |
| SI 2 vs. Colon 1 | ns | 0.1551 | ns | 0.9975 | ns | 0.9994 | * | 0.0318 |
| SI 2 vs. Colon 2 | ns | 0.8406 | ns | 0.9483 | ns | 0.9056 | ns | 0.4899 |
| SI 2 vs. Colon 3 | ns | >0.9999 | ns | 0.9963 | ns | 0.9561 | ns | >0.9999 |
| SI 3 vs. SI 4 | ns | >0.9999 | ns | >0.9999 | ns | >0.9999 | ns | >0.9999 |
| SI 3 vs. SI 5 | ns | 0.4868 | ns | >0.9999 | ns | 0.9984 | ns | >0.9999 |
| SI 3 vs. SI 6 | ns | >0.9999 | ns | 0.9999 | ns | 0.5623 | ns | 0.9907 |
| SI 3 vs. Cecum | *** | 0.0007 | **** | <0.0001 | **** | <0.0001 | **** | <0.0001 |
| SI 3 vs. Colon 1 | ns | 0.1815 | ns | 0.9312 | ns | 0.9927 | * | 0.0182 |
| SI 3 vs. Colon 2 | ns | 0.8728 | ns | 0.7044 | ns | 0.7692 | ns | 0.3713 |
| SI 3 vs. Colon 3 | ns | >0.9999 | ns | 0.918 | ns | 0.8626 | ns | >0.9999 |
| SI 4 vs. SI 5 | ns | 0.4769 | ns | >0.9999 | ns | >0.9999 | ns | >0.9999 |
| SI 4 vs. SI 6 | ns | >0.9999 | ns | 0.9999 | ns | 0.7759 | ns | 0.9943 |
| SI 4 vs. Cecum | *** | 0.0007 | **** | <0.0001 | **** | <0.0001 | **** | <0.0001 |
| SI 4 vs. Colon 1 | ns | 0.1758 | ns | 0.9273 | ns | 0.9996 | * | 0.0226 |
| SI 4 vs. Colon 2 | ns | 0.8665 | ns | 0.6958 | ns | 0.9191 | ns | 0.4145 |
| SI 4 vs. Colon 3 | ns | >0.9999 | ns | 0.9136 | ns | 0.9639 | ns | >0.9999 |
| SI 5 vs. SI 6 | ns | 0.697 | ns | >0.9999 | ns | 0.9766 | ns | 0.9994 |
| SI 5 vs. Cecum | ns | 0.4798 | **** | <0.0001 | *** | 0.0006 | **** | <0.0001 |
| SI 5 vs. Colon 1 | ns | >0.9999 | ns | 0.95 | ns | >0.9999 | * | 0.0472 |
| SI 5 vs. Colon 2 | ns | >0.9999 | ns | 0.7497 | ns | 0.9974 | ns | 0.5829 |
| SI 5 vs. Colon 3 | ns | 0.4723 | ns | 0.9393 | ns | 0.9996 | ns | >0.9999 |
| SI 6 vs. Cecum | ** | 0.0024 | **** | <0.0001 | ns | 0.0503 | **** | <0.0001 |
| SI 6 vs. Colon 1 | ns | 0.3339 | ns | 0.9993 | ns | 0.9929 | ns | 0.3149 |
| SI 6 vs. Colon 2 | ns | 0.9648 | ns | 0.9742 | ns | >0.9999 | ns | 0.9667 |
| SI 6 vs. Colon 3 | ns | >0.9999 | ns | 0.999 | ns | >0.9999 | ns | 0.9961 |
| Cecum vs. Colon 1 | ns | 0.8301 | **** | <0.0001 | ** | 0.0013 | *** | 0.0004 |
| Cecum vs. Colon 2 | ns | 0.1477 | **** | <0.0001 | * | 0.0196 | **** | <0.0001 |
| Cecum vs. Colon 3 | *** | 0.0007 | **** | <0.0001 | * | 0.0109 | **** | <0.0001 |
| Colon 1 vs. Colon 2 | ns | 0.9869 | ns | >0.9999 | ns | 0.9996 | ns | 0.9828 |
| Colon 1 vs. Colon 3 | ns | 0.1732 | ns | >0.9999 | ns | >0.9999 | * | 0.0262 |
| Colon 2 vs. Colon 3 | ns | 0.8635 | ns | >0.9999 | ns | >0.9999 | ns | 0.4462 |

**Supplementary Table 5:** Tukey`s multiple comparisons (GraphPad Prism Version 11; [www.graphpad.com](http://www.graphpad.com)) between intestinal regions (within each group) of 250 kDa-FITC dextran distribution in the GI tract of female mice after 180 min of oral administration.

| **FEMALE**  **(Exp 180 min)** | **CONTROL** | | **SMM** | | **NSM** | | **MFM** | |
| --- | --- | --- | --- | --- | --- | --- | --- | --- |
| Intestinal regions |  | P-VALUE |  | P-VALUE |  | P-VALUE |  | P-VALUE |
| Sto vs. SI 1 | ns | >0.9999 | ns | >0.9999 | ns | >0.9999 | ns | >0.9999 |
| Sto vs. SI 2 | ns | >0.9999 | ns | >0.9999 | ns | >0.9999 | ns | >0.9999 |
| Sto vs. SI 3 | ns | >0.9999 | ns | >0.9999 | ns | >0.9999 | ns | >0.9999 |
| Sto vs. SI 4 | ns | >0.9999 | ns | >0.9999 | ns | >0.9999 | ns | >0.9999 |
| Sto vs. SI 5 | ns | 0.1907 | ns | >0.9999 | ns | >0.9999 | ns | 0.9997 |
| Sto vs. SI 6 | **** | <0.0001 | ns | 0.8971 | ns | 0.7356 | ns | 0.1955 |
| Sto vs. Cecum | **** | <0.0001 | **** | <0.0001 | **** | <0.0001 | **** | <0.0001 |
| Sto vs. Colon 1 | ns | 0.9975 | * | 0.0193 | ns | 0.0596 | ns | 0.5003 |
| Sto vs. Colon 2 | ns | >0.9999 | ns | 0.4917 | ns | 0.4702 | ns | 0.6304 |
| Sto vs. Colon 3 | ns | >0.9999 | ns | >0.9999 | ns | >0.9999 | ns | >0.9999 |
| SI 1 vs. SI 2 | ns | >0.9999 | ns | >0.9999 | ns | >0.9999 | ns | >0.9999 |
| SI 1 vs. SI 3 | ns | >0.9999 | ns | >0.9999 | ns | >0.9999 | ns | >0.9999 |
| SI 1 vs. SI 4 | ns | >0.9999 | ns | >0.9999 | ns | >0.9999 | ns | >0.9999 |
| SI 1 vs. SI 5 | ns | 0.2864 | ns | >0.9999 | ns | >0.9999 | ns | 0.9995 |
| SI 1 vs. SI 6 | *** | 0.0001 | ns | 0.8892 | ns | 0.7198 | ns | 0.174 |
| SI 1 vs. Cecum | *** | 0.0002 | **** | <0.0001 | **** | <0.0001 | **** | <0.0001 |
| SI 1 vs. Colon 1 | ns | 0.9997 | * | 0.018 | ns | 0.0556 | ns | 0.4643 |
| SI 1 vs. Colon 2 | ns | >0.9999 | ns | 0.4776 | ns | 0.4531 | ns | 0.594 |
| SI 1 vs. Colon 3 | ns | >0.9999 | ns | >0.9999 | ns | >0.9999 | ns | >0.9999 |
| SI 2 vs. SI 3 | ns | >0.9999 | ns | >0.9999 | ns | >0.9999 | ns | >0.9999 |
| SI 2 vs. SI 4 | ns | >0.9999 | ns | >0.9999 | ns | >0.9999 | ns | >0.9999 |
| SI 2 vs. SI 5 | ns | 0.2004 | ns | >0.9999 | ns | >0.9999 | ns | 0.9995 |
| SI 2 vs. SI 6 | **** | <0.0001 | ns | 0.8867 | ns | 0.7587 | ns | 0.1777 |
| SI 2 vs. Cecum | *** | 0.0001 | **** | <0.0001 | **** | <0.0001 | **** | <0.0001 |
| SI 2 vs. Colon 1 | ns | 0.998 | * | 0.0176 | ns | 0.0662 | ns | 0.4707 |
| SI 2 vs. Colon 2 | ns | >0.9999 | ns | 0.4732 | ns | 0.4961 | ns | 0.6006 |
| SI 2 vs. Colon 3 | ns | >0.9999 | ns | >0.9999 | ns | >0.9999 | ns | >0.9999 |
| SI 3 vs. SI 4 | ns | >0.9999 | ns | >0.9999 | ns | >0.9999 | ns | >0.9999 |
| SI 3 vs. SI 5 | ns | 0.2503 | ns | >0.9999 | ns | >0.9999 | ns | 0.9996 |
| SI 3 vs. SI 6 | **** | <0.0001 | ns | 0.9144 | ns | 0.759 | ns | 0.1869 |
| SI 3 vs. Cecum | *** | 0.0002 | **** | <0.0001 | **** | <0.0001 | **** | <0.0001 |
| SI 3 vs. Colon 1 | ns | 0.9993 | * | 0.0225 | ns | 0.0663 | ns | 0.4861 |
| SI 3 vs. Colon 2 | ns | >0.9999 | ns | 0.5255 | ns | 0.4965 | ns | 0.6162 |
| SI 3 vs. Colon 3 | ns | >0.9999 | ns | >0.9999 | ns | >0.9999 | ns | >0.9999 |
| SI 4 vs. SI 5 | ns | 0.5247 | ns | >0.9999 | ns | >0.9999 | ns | 0.9998 |
| SI 4 vs. SI 6 | *** | 0.0005 | ns | 0.9172 | ns | 0.8743 | ns | 0.204 |
| SI 4 vs. Cecum | ** | 0.0011 | **** | <0.0001 | **** | <0.0001 | **** | <0.0001 |
| SI 4 vs. Colon 1 | ns | >0.9999 | * | 0.0231 | ns | 0.1187 | ns | 0.514 |
| SI 4 vs. Colon 2 | ns | >0.9999 | ns | 0.5313 | ns | 0.651 | ns | 0.6439 |
| SI 4 vs. Colon 3 | ns | >0.9999 | ns | >0.9999 | ns | >0.9999 | ns | >0.9999 |
| SI 5 vs. SI 6 | ns | 0.4174 | ns | 0.96 | ns | 0.9078 | ns | 0.6512 |
| SI 5 vs. Cecum | ns | 0.5399 | **** | <0.0001 | **** | <0.0001 | **** | <0.0001 |
| SI 5 vs. Colon 1 | ns | 0.779 | * | 0.0383 | ns | 0.1462 | ns | 0.9243 |
| SI 5 vs. Colon 2 | ns | 0.5404 | ns | 0.6454 | ns | 0.7082 | ns | 0.9681 |
| SI 5 vs. Colon 3 | ns | 0.2325 | ns | >0.9999 | ns | >0.9999 | ns | 0.9999 |
| SI 6 vs. Cecum | ns | >0.9999 | **** | <0.0001 | **** | <0.0001 | ** | 0.0014 |
| SI 6 vs. Colon 1 | ** | 0.0024 | ns | 0.6541 | ns | 0.9652 | ns | >0.9999 |
| SI 6 vs. Colon 2 | *** | 0.0006 | ns | 0.9999 | ns | >0.9999 | ns | 0.9998 |
| SI 6 vs. Colon 3 | **** | <0.0001 | ns | 0.9289 | ns | 0.9648 | ns | 0.2191 |
| Cecum vs. Colon 1 | ** | 0.0047 | *** | 0.0001 | ** | 0.003 | *** | 0.0002 |
| Cecum vs. Colon 2 | ** | 0.0012 | **** | <0.0001 | **** | <0.0001 | **** | <0.0001 |
| Cecum vs. Colon 3 | *** | 0.0001 | **** | <0.0001 | **** | <0.0001 | **** | <0.0001 |
| Colon 1 vs. Colon 2 | ns | >0.9999 | ns | 0.9624 | ns | 0.9975 | ns | >0.9999 |
| Colon 1 vs. Colon 3 | ns | 0.999 | * | 0.026 | ns | 0.2361 | ns | 0.5375 |
| Colon 2 vs. Colon 3 | ns | >0.9999 | ns | 0.5576 | ns | 0.8342 | ns | 0.6667 |

**References**

Faggin, S., V. Caputi, A. Tietto, M.C. Giron. 2026. Immunohistochemical Localization and Anatomical Distribution of Enteric Glia Cells in Enteric Nervous System. Methods Mol Biol 2971: 7-19

Bossolani, G.D.P., I. Pintelon, J.D. Detrez, R. Buckinx, S. Thys, J.N. Zanoni, W.H. De Vos, J.P. Timmermans. 2019. Comparative analysis reveals Ce3D as optimal clearing method for in toto imaging of the mouse intestine. Neurogastroenterol Motil 31: e13560.

Beebe, N.L., J.W. Young, J.G. Mellott, B.R. Schofield. 2016. Extracellular Molecular Markers and Soma Size of Inhibitory Neurons: Evidence for Four Subtypes of GABAergic Cells in the Inferior Colliculus. J Neurosci 36: 3988-3999.

Faggin, S., S. Cerantola, A. Bosi, C. Giaroni, E. Napoli, E.V. Savarino, M. Contran, A. Porzionato, C. Pellegrini, L. Antonioli, V. Caputi, M.C. Giron. 2025a. Small Intestine Neuromuscular Dysfunctions and Neurogliopathy in a Mouse Model of High-Fat Diet-Induced Obesity: Involvement of Toll-Like Receptor 4. Int J Mol Sci 26.

Frith, M.E., P.C. Kashyap, D.R. Linden, B. Theriault, E.B. Chang. 2024. Microbiota-dependent early-life programming of gastrointestinal motility. iScience 27: 110895.
